# Functional Characterization of a *Bacillus*-Derived Novel Broad-Spectrum Antifungal Lipopeptide Variant against Candida tropicalis and Candida auris and Unravelling Its Mode of Action

**DOI:** 10.1128/spectrum.01583-22

**Published:** 2023-02-06

**Authors:** Swetha Ramesh, Madhuri Madduri, Shivaprakash M. Rudramurthy, Utpal Roy

**Affiliations:** a Department of Biological Sciences, BITS Pilani K.K. Birla Goa Campus, Goa, India; b Department of Medical Microbiology, Post Graduate Institute of Medical Education & Research (PGIMER), Chandigarh, India; University of Debrecen

**Keywords:** antifungal, CLSM, flow cytometry, lipopeptide, time-kill assay, antifungal agents, antifungal susceptibility testing, antifungal therapy

## Abstract

Limited treatment options, recalcitrance, and resistance to existing therapeutics encourage the discovery of novel antifungal leads for alternative therapeutics. Antifungal lipopeptides have emerged as potential candidates for developing new and alternative antifungal therapies. In our previous studies, we isolated and identified the lipopeptide variant AF_4_ and purified it to homogeneity via chromatography from the cell-free supernatant of Bacillus subtilis. AF_4_ was found to have broad-spectrum antifungal activity against more than 110 fungal isolates. In this study, we found that clinical isolates of Candida tropicalis and Candida auris exposed to AF_4_ exhibited low MICs of 4 to 8 mg/L. Time-kill assays indicated the *in vitro* pharmacodynamic potential of AF_4_. Biocompatibility assays demonstrated ~75% cell viability at 8 mg/L of AF_4_, indicating the lipopeptide’s minimally cytotoxic nature. In lipopeptide-treated C. tropicalis and C. auris cells, scanning electron microscopy revealed damage to the cell surface, while confocal microscopy with acridine orange(AO)/propidium iodide (PI) and FUN-1 indicated permeabilization of the cell membrane, and DNA damage upon DAPI (4′,6-diamidino-2-phenylindole) staining. These observations were corroborated using flow cytometry (FC) in which propidium iodide, 2′,7′-dichlorodihydrofluorescein diacetate (DCFH-DA), and rhodamine 123 (Rh123) staining of cells treated with AF_4_ revealed loss of membrane integrity, increased reactive oxygen species (ROS) production, and mitochondrial membrane dysfunction, respectively. Membrane perturbation was also observed in the 1,6-diphenyl-1,3,5-hexatriene (DPH) fluorescence study and the interaction with ergosterol was observed by an ergosterol binding assay. Decreased membrane dipole potential also indicated the probable binding of lipopeptide to the cell membrane. Collectively, these findings describe the mode of action of AF_4_ against fungal isolates by membrane disruption and ROS generation, demonstrating its antifungal potency.

**IMPORTANCE** C. tropicalis is a major concern for candidiasis in India and C. auris has emerged as a resistant yeast causing difficult-to-treat infections. Currently, amphotericin B (AMB) and 5-flucytosine (5-FC) are the main therapeutics for systemic fungal infections; however, the nephrotoxicity of AMB and resistance to 5-FC is a serious concern. Antifungal lead molecules with low adverse effects are the need of the hour. In this study, we briefly describe the antifungal potential of the AF_4_ lipopeptide and its mode of action using microscopy, flow cytometry, and fluorescence-based assays. Our investigation reveals the basic mode of action of the investigated lipopeptide. This lipopeptide with broad-spectrum antifungal potency is apparently membrane-active, and there is a smaller chance that organisms exposed to such a compound will develop drug resistance. It could potentially act as a lead molecule for the development of an alternative antifungal agent to combat candidiasis.

## INTRODUCTION

Candida albicans, a dimorphic fungus which causes either superficial (oral, vaginal, and mucocutaneous) or deep-seated (e.g., acute disseminated candida septicemia) candidiasis, is one of the leading opportunistic fungal pathogens in immunocompromised patients (1). The distribution of *Candida* species has experienced an increase in non-*albicans* species such as Candida glabrata, Candida parasilopsis, Candida tropicalis, and Candida krusei. In India, C. tropicalis is the species most commonly isolated from candidemia cases, followed by C. albicans (2). Non-*albicans Candida* (NAC) can cause infections sporadically, often complicating the management of candidiasis, because candidiasis can be recalcitrant to treatment or become resistant (3–5).

Infections caused by NAC species have been on the rise in India as well, with C. tropicalis prevailing as the leading pathogen, along with reports of Candida auris isolates in several intensive care units (6). NAC species were recognized as the major causative agent of opportunistic fungal infections, and studies conducted over the last few decades have revealed a steady shift from a predominance of C. albicans to NAC such as C. tropicalis and C. glabrata (4, 7, 8). To add to these woes, C. auris isolates have emerged as important drug-resistant nosocomial pathogens in various clinical settings (9). In this regard, the Centers for Disease Control and Prevention have raised concerns and cautioned that emerging multidrug-resistant C. auris could pose a global threat to public health (10).

New antifungals are needed to combat the emergence of resistance to existing therapies (11, 12). Effective fungicides can potentially prevent the development of antifungal resistance. Therefore, finding new fungicidal lead compounds is an essential task for the development of ideal antifungal drugs (13, 14). The genus *Bacillus* is known to produce several antimicrobial compounds, including polyketides, non-ribosomally synthesized peptides, and bacteriocins (15, 16). Among these, peptide derivatives have received more attention due to their antifungal properties (13, 17). Antimicrobial peptide-based design of compounds has gained attention because of the potent antifungal agents form multimeric pores in the cell membranes, leading to cell lysis or interaction with RNA or DNA after penetration into the cell.

In our previous studies (13), we isolated and purified antifungal lead molecules from wild-type Bacillus subtilis using an optimized multistep process. Using reversed-phase high-performance liquid chromatography (RP-HPLC), we identified three antifungal lead molecules with broad spectrum activity against 81 fungal isolates of *Candida* and Cryptococcus species and 11 isolates of filamentous fungi (13). Using electrospray ionization fourier transform ion cyclotron resonance mass spectrometry (ESI-FT-ICR-MS) and gas chromatography-mass spectrometry (GC-MS), we identified the lead molecule as a lipopeptide with an *m/z* of 1,071.4. The antifungal lipopeptides also showed low hemolysis and anti-biofilm activity (13). Of the three lipopeptides, AF_4_ was found to have the best activity and low cytotoxicity and was chosen for further characterization.

The present study was aimed at functional characterization of the AF_4_ lipopeptide variant against C. tropicalis and C. auris and studying the mode of action of the reversed-phase HPLC-purified antifungal lipopeptide variant AF_4_ (13, 14).

## RESULTS

The antifungal lipopeptide variant AF_4_ was extracted and purified using a multistep process as shown in Fig. S1. Throughout the entire study, the RP-HPLC-purified AF_4_-lipopeptide variant (*m/z* 1,071.4, Fig. S2) (13, 14) was used for functional characterization.

### Antifungal susceptibility of NAC.

The MICs and MFCs (minimum fungicidal concentrations) of RP-HPLC-purified antifungal variant AF_4_ against the clinical isolates of Candida tropicalis ATCC 750 and Candida auris IL-3331 ranged between 4 and 8 mg/L (Table S1). The MICs of C. tropicalis and C. auris were 4 mg/L (Table S1). The MICs for amphotericin B (AMB) were 1 and 2 mg/L for C. tropicalis and C. auris, respectively (Table S1).

### Time-kill kinetics.

**FIG 1:**
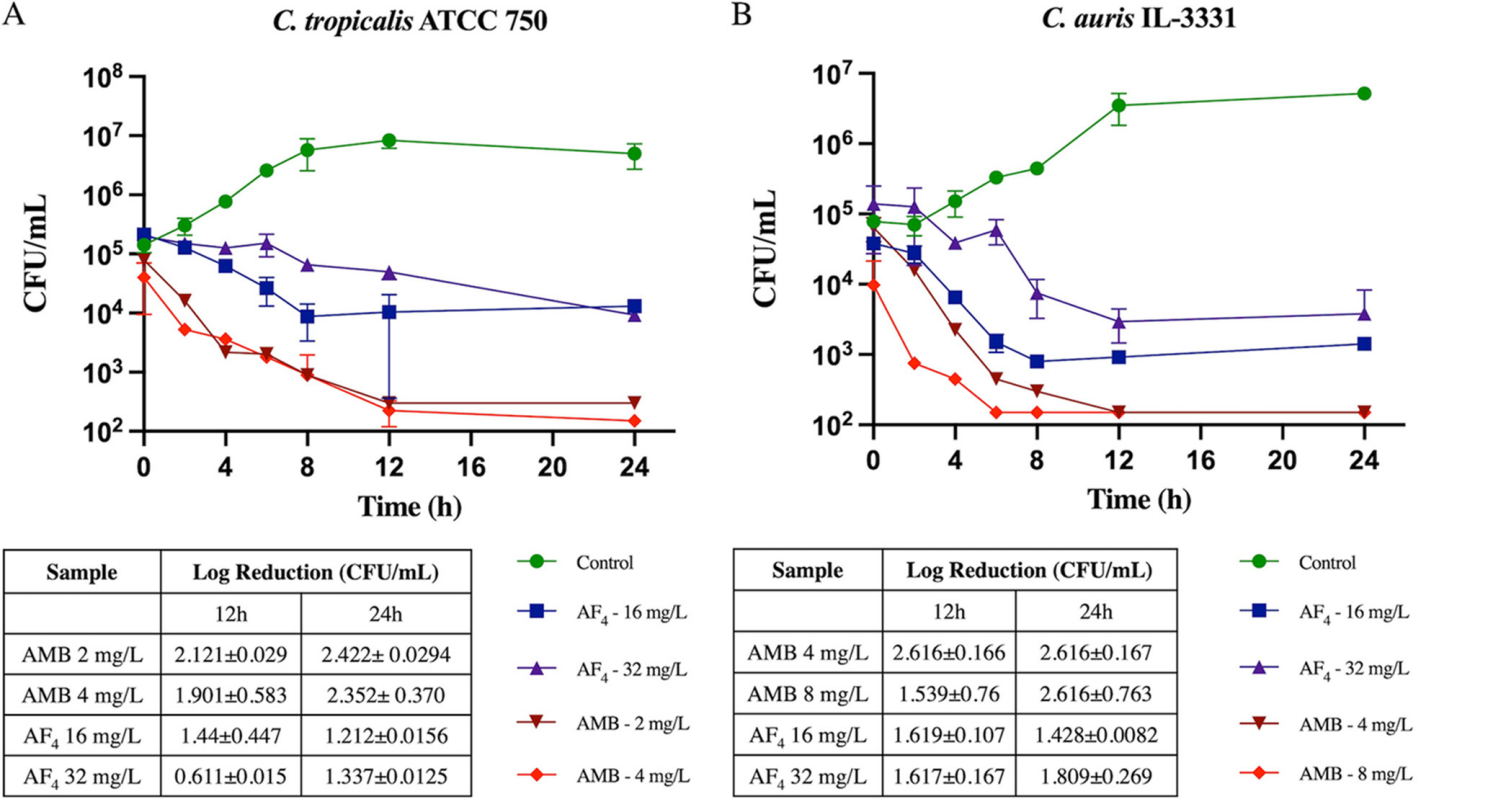
Time-killing kinetics study of AF_4_ and amphotericin B (AMB). (A) AF_4_ and AMB against Candida tropicalis ATCC 750. (B) AF_4_ and AMB against Candida auris IL-3331. Log-reduction values of each treatment at 12 and 24 h are shown in the inset. Each data point represents the mean result ± standard deviation (SD) of experiments performed in duplicate. One-way analysis of variance (ANOVA) followed by Dunnett’s test showed *P < *0.05.

Time-kill kinetics were performed to determine the effects of different concentrations of the antifungal compound over time. The antifungal lipopeptide AF_4_ at concentrations of 16 and 32 mg/L produced a significant decrease in CFU/mL over a period of 24 h. The log reduction in CFU/mL (Fig. 1A and B, insets) and curves representing the reduction in CFU/mL are shown in the graphs in Fig. 1A and B.

C. tropicalis treated with AF_4_ at 16 and 32 mg/L showed percentage reductions of 93.87% and 95.40%, respectively, after 24 h. Interestingly, after 12 h, AF_4_ produced reductions of 96.43% and 75.036% at 16 and 32 mg/L, respectively (Fig. 1A); this reduction of >50% was significant because it indicates the manifestation of antifungal action of AF_4_ from 12 h onwards.

The time-kill kinetics of AF_4_ against C. auris also showed promising results after 24 h of incubation (Fig. 1B). The percentage reduction in CFU/mL observed after treatment with 16 mg/L AF_4_ was 96.27%, and that after treatment with 32 mg/L AF_4_ was determined to be 98.29%. The corresponding percentage reductions at 12 h were as high as 97.56% at 16 mg/L and 97.49% at 32 mg/L, respectively, showing near-fungicidal activity at 12 h.

Time-kill curves were also generated for AMB against both C. tropicalis and C. auris (Fig. 1A and B). Against C. auris after 24 h, the percentage reduction was 99.74% when treated with 4 mg/L AMB and 99.9% when treated with 8 mg/L AMB. Similarly, AMB-treated C. tropicalis showed a 99.6% reduction at 2 mg/L and a 99.47% reduction at 4 mg/L, respectively.

### Biocompatibility analysis of AF_4_ with mammalian cell lines.

**FIG 2:**
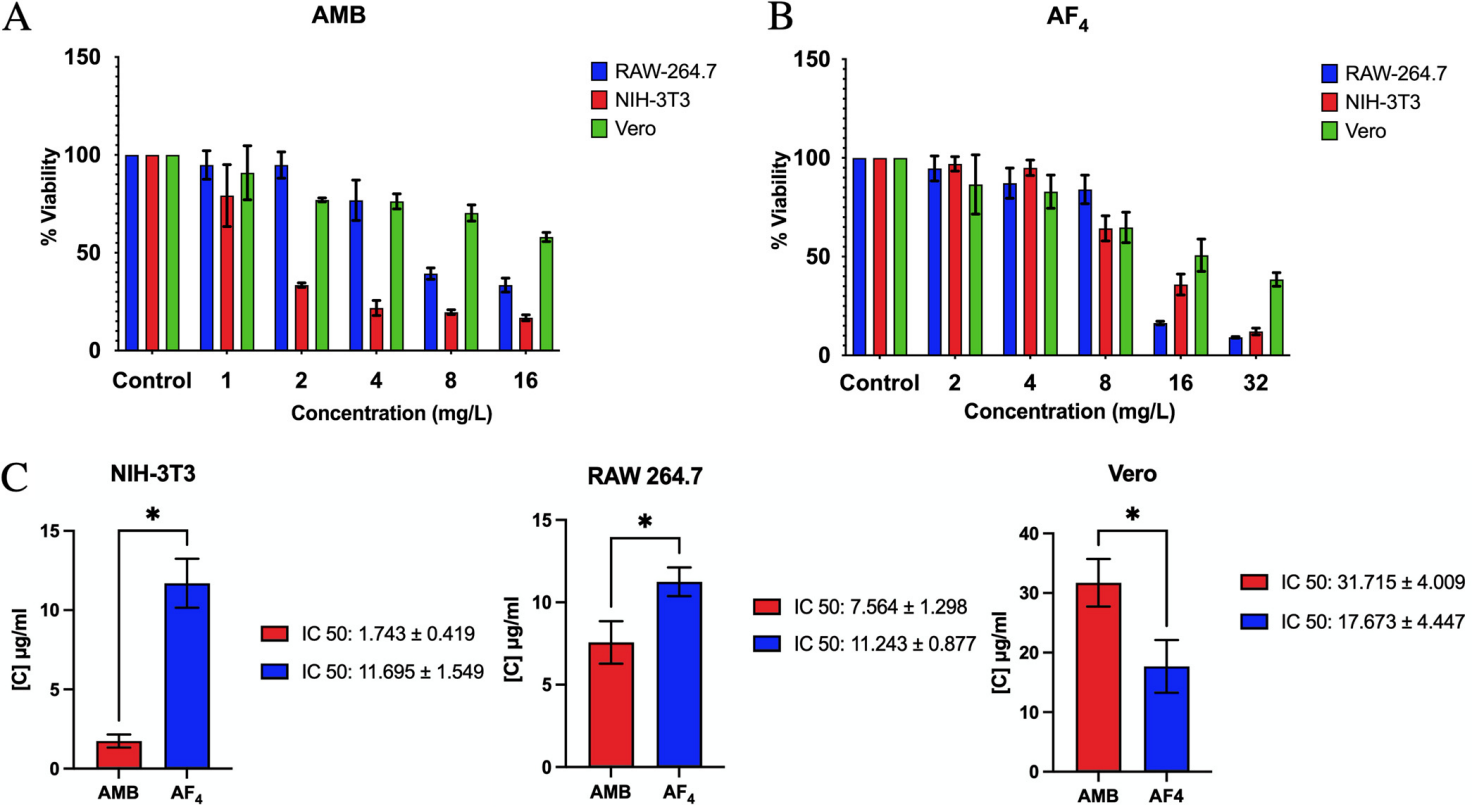
Biocompatibility of antifungals AMB and AF_4_. (A) Percentage of viability of RAW 264.7, NIH 3T3, and Vero cells treated with AMB (1 to 16 mg/L). (B) Percentage of viability of RAW 264.7, NIH 3T3, and Vero cells treated with AF_4_ (2 to 32 mg/L). (C) Comparative analysis of the IC_50_ (50% inhibitory concentration) values of AMB and AF_4_ for each cell line. Each data point represents mean result ± SD; *P < *0.05 indicates a statistically significant difference by unpaired *t* test.

Using an 3-(4,5-dimethylthiazol-2-yl)-2,5-diphenyltetrazolium bromide (MTT) assay, we tested the biocompatibility of the antifungal variant AF_4_ by assessing the percentage of cells that remained viable in the selected cell lines RAW 264.7, NIH 3T3, and Vero post-treatment. In AMB-treated cells, viability declined sharply for the RAW 264.7 and NIH 3T3 cells treated with 8 mg/L AF_4_ (Fig. 2A). In all cell lines tested, more than 50% of cells were viable upon treatment with up to 8 mg/L of AF_4_ lipopeptide (Fig. 2B). AF_4_ only exhibited toxicity at a high concentration of 32 mg/L. The 50% inhibitory concentration (IC_50_) values (Fig. 2C) of all cell lines were found to be significantly higher than the MFC values of both AMB and AF_4_.

### Visualization of cell membrane damage.

**FIG 3:**
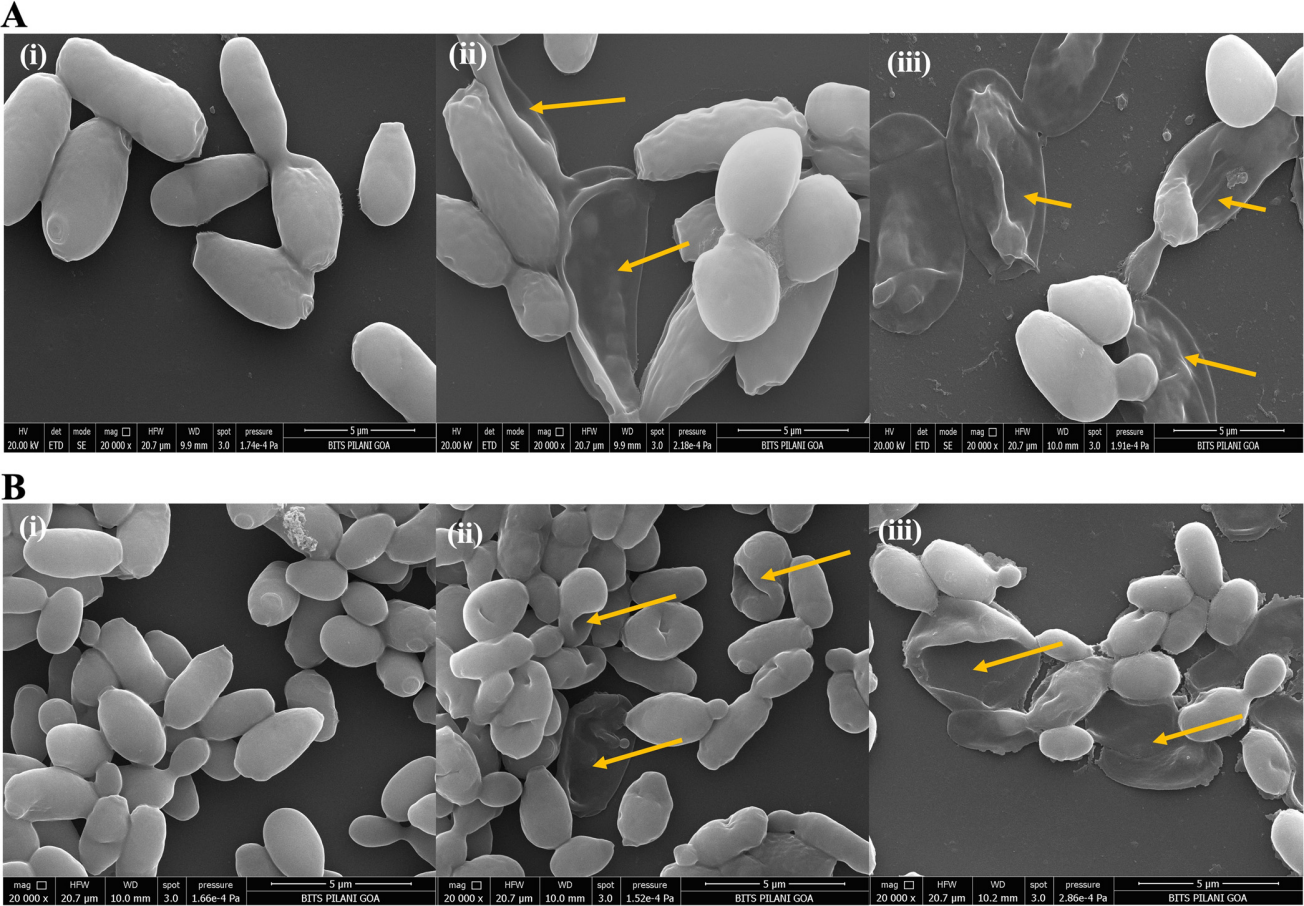
Scanning electron micrographs exhibiting ultrastructure alterations. Damage to the ultrastructure of the cell and membrane damage are shown. (A) C. tropicalis ATCC 750 cells (i) without treatment, (ii) treated with AMB (2 mg/L), or (iii) treated with AF_4_ (8 mg/L). (B) C. auris IL-3331 cells (i) without treatment, (ii) treated with AMB (4 mg/L), and (iii) treated with AF_4_ (8 mg/L).

Scanning electron micrographs (Fig. 3A and Fig. 3B) of C. tropicalis and C. auris revealed that treatment with a standard antifungal and the novel lipopeptide AF_4_ significantly damaged and altered the ultrastructure of the cells. Untreated cells maintained their rounded or oval shape with a smooth surface and bud-scars, indicating that they were healthy, whereas cells treated with antifungal drugs showed characteristic aggregation and membrane damage, discerned as surface deformities, dimples, or cavities, leading to loss of cellular architecture (Fig. 3A and Fig. S3). Similar deformities were observed in AMB-treated cells of both strains (Fig. 3A and B, panel ii). AF_4_-treated C. tropicalis cells (Fig. S3A) also exhibited occasional membrane blebs on the cell surface in addition to complete ultrastructural collapse with loss of shape.

### Confocal microscopy of *Candida* cells.

**FIG 4:**
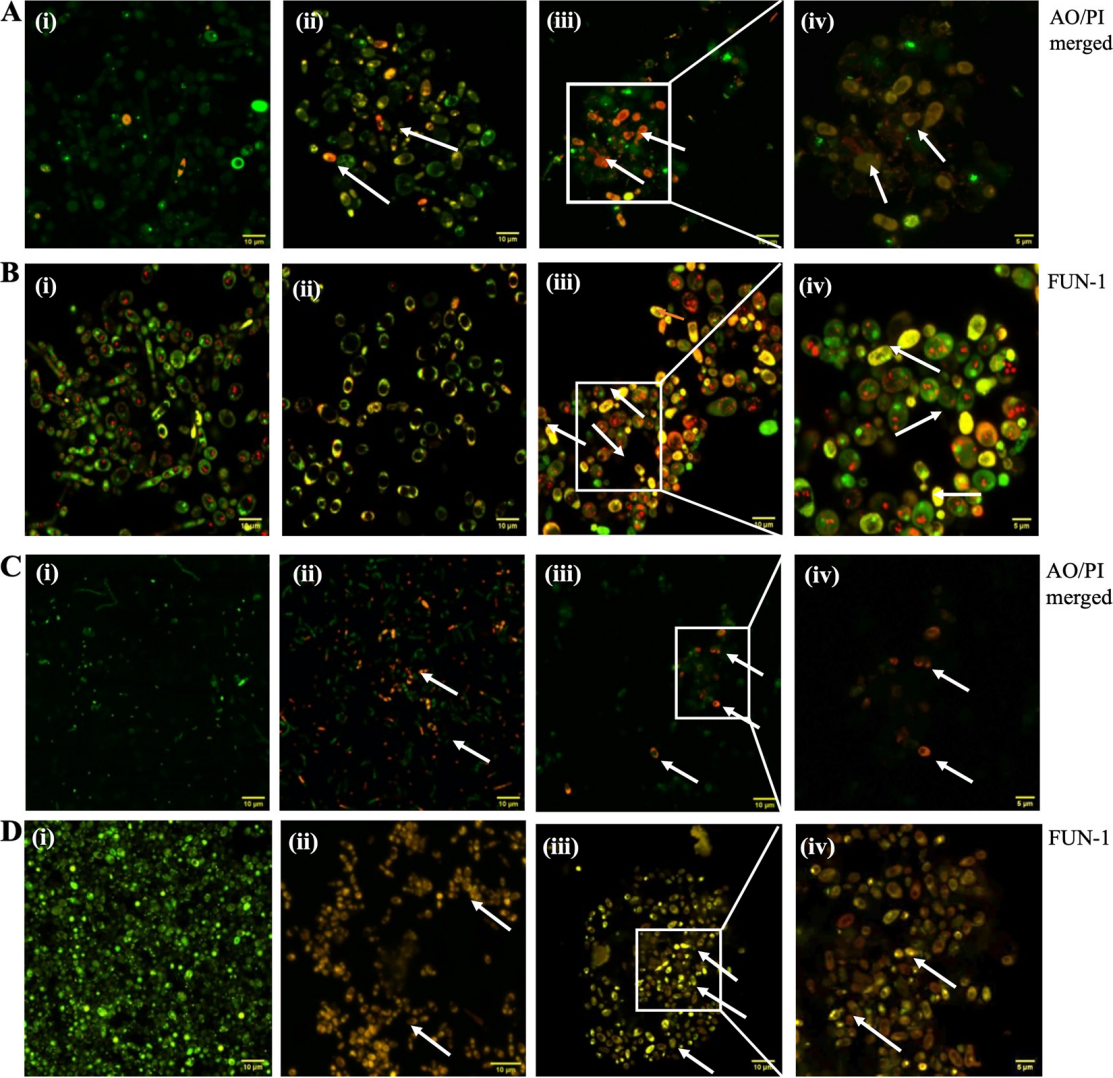
Confocal microscopy images of cells stained with acridine orange (AO)/propidium iodide (PI) and with FUN-1. (A and B) C. tropicalis ATCC 750 cells (i) without treatment, (ii) treated with AMB (2 mg/L), and treated with AF_4_ (8 mg/L) at (iii) ×120 and (iv) ×200 magnification, stained with AO/PI and FUN-1, respectively. (C and D) C. auris IL-3331 cells (i) without treatment, (ii) treated with AMB (4 mg/L), and treated with AF_4_ (8 mg/L) at (iii) ×120 and (iv) ×200 magnification, stained with AO/PI. In panels A (ii to iv) and C (ii to iv), AMB and AF_4_ treated cells are orange-yellow in color due to the combined fluorescence of AO and PI, visualized only in the case of damaged cells. In FUN-1 panels, the treated cells seen in panels B (ii to iv) and D (ii to iv) lack reddish cylindrical intravacuolar structures and appear yellow-green in color, indicating the loss of metabolic activity.

The cell membrane permeability was assessed using acridine orange (AO), a cell-permeable stain that stains both live and dead cells and is uniformly visible as green fluorescence. The membrane-impermeant fluorescent dye propidium iodide (PI) stains only cells with compromised membrane integrity and is visualized as red fluorescence. The absence of red fluorescence in the control panels (Fig. 4A and C) indicates a lack of cell membrane damage and therefore exclusion of PI, whereas cells treated with AMB and AF_4_ exhibited cell membrane damage and PI uptake, visible as orange-red or orange-yellow fluorescence (Fig. 4A and C).

To visualize the effect of antifungals on the metabolic state of C. tropicalis and C. auris cells, we used a FUN-1 viability stain. FUN-1 is a two-color fluorescent probe which fluoresces diffused green and is transported to vacuoles to form red fluorescent, cylindrical intravacuolar structures (CIVS) in fungal cells which retain their membrane integrity and metabolic activity, whereas dead cells fluoresce bright yellow-green with no discernible red fluorescent CIVS (18). As shown in Fig. 4B and D, untreated cells remained viable and showed the formation of red fluorescent CIVS, while cells treated with AMB and AF_4,_ respectively, showed yellow-green fluorescence, indicating the loss of metabolic activity.

### Ergosterol binding by AF_4_.

Ergosterol is the main membrane sterol of fungal cells. The ability of AF_4_ to cause membrane destabilization can be identified by its ability to bind to exogenous ergosterol added to the cell suspension. The complexation of lipopeptide with membrane ergosterol is impeded in the presence of exogenous ergosterol, resulting in a subsequent MIC increase (19). In the presence of exogenous ergosterol at 100, 200, and 400 μg/mL, the MIC of AF_4_ increased 16-fold, from 4 to >64 mg/L (Table 1) for both C. tropicalis and C. auris. A similar increase in MIC values was observed in *Candida* cells treated with AMB (Table 1). These results indicate that AF_4_ may exert its function by binding to membrane ergosterol.

**TABLE 1:** MICs of AF_4_ and AMB against Candida tropicalis ATCC 750 and Candida auris IL-3331 in the presence and absence of ergosterol

Antifungal	C. tropicalis ATCC 750				C. auris IL-3331			
	Ergosterol				Ergosterol			
	MIC in Absence	MIC in Presence (μg/mL)			MIC in Absence	MIC in Presence (μg/mL)		
		100	200	400		100	200	400
AMB^*a*^	1	2	8	16	2	2	8	16
AF_4_	4	>64	>64	>64	4	>64	>64	>64

a Amphotericin B.

### Changes to membrane dynamics using DPH fluorescence.

**FIG 5:**
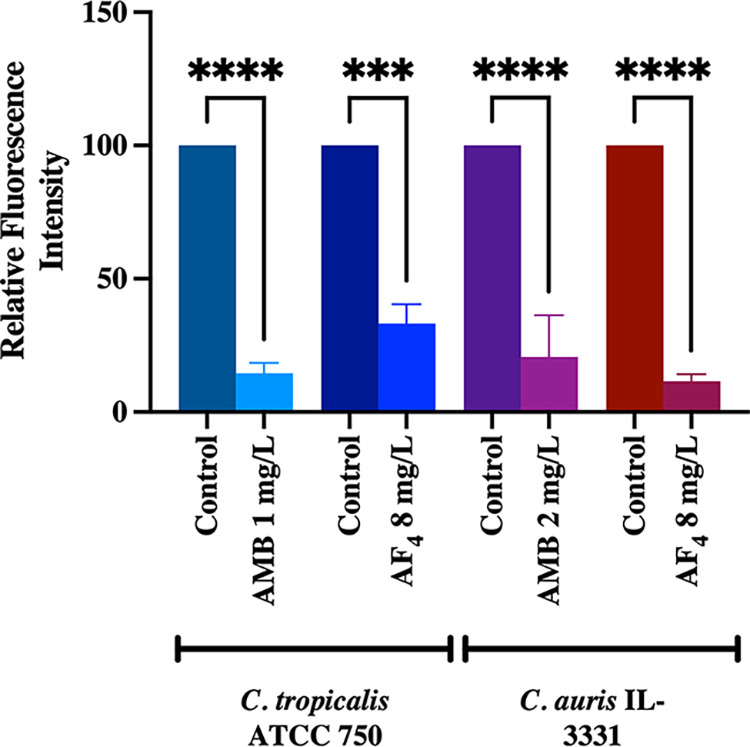
DPH (1,6-diphenyl-1,3,5-hexatriene) fluorescence estimation. Relative fluorescence intensity of DPH-labeled cells after treatment with AMB at respective MICs and AF_4_ at 8 mg/L. Both experiments were performed in duplicate; assessment with one-way ANOVA resulted in *P* < 0.0001.

1,6-diphenyl 1.3.5-hexatriene (DPH) fluorescence is used as an indicator of stable membrane dynamics. Untreated cells showed high fluorescence intensity, whereas cells treated with AMB at 1× MIC and AF_4_ at 8 mg/L showed a significant decrease in fluorescence intensity due to membrane perturbation (Fig. 5). C. tropicalis cells treated with AMB and AF_4_ showed 44.02% and 33.21% intensity, while C. auris cells showed 20.67% and 18.02% intensity respectively, compared to the intensity of fluorescence signal obtained for the respective untreated cells (Fig. 5).

### Evaluation of membrane dipole potential.

**FIG 6:**
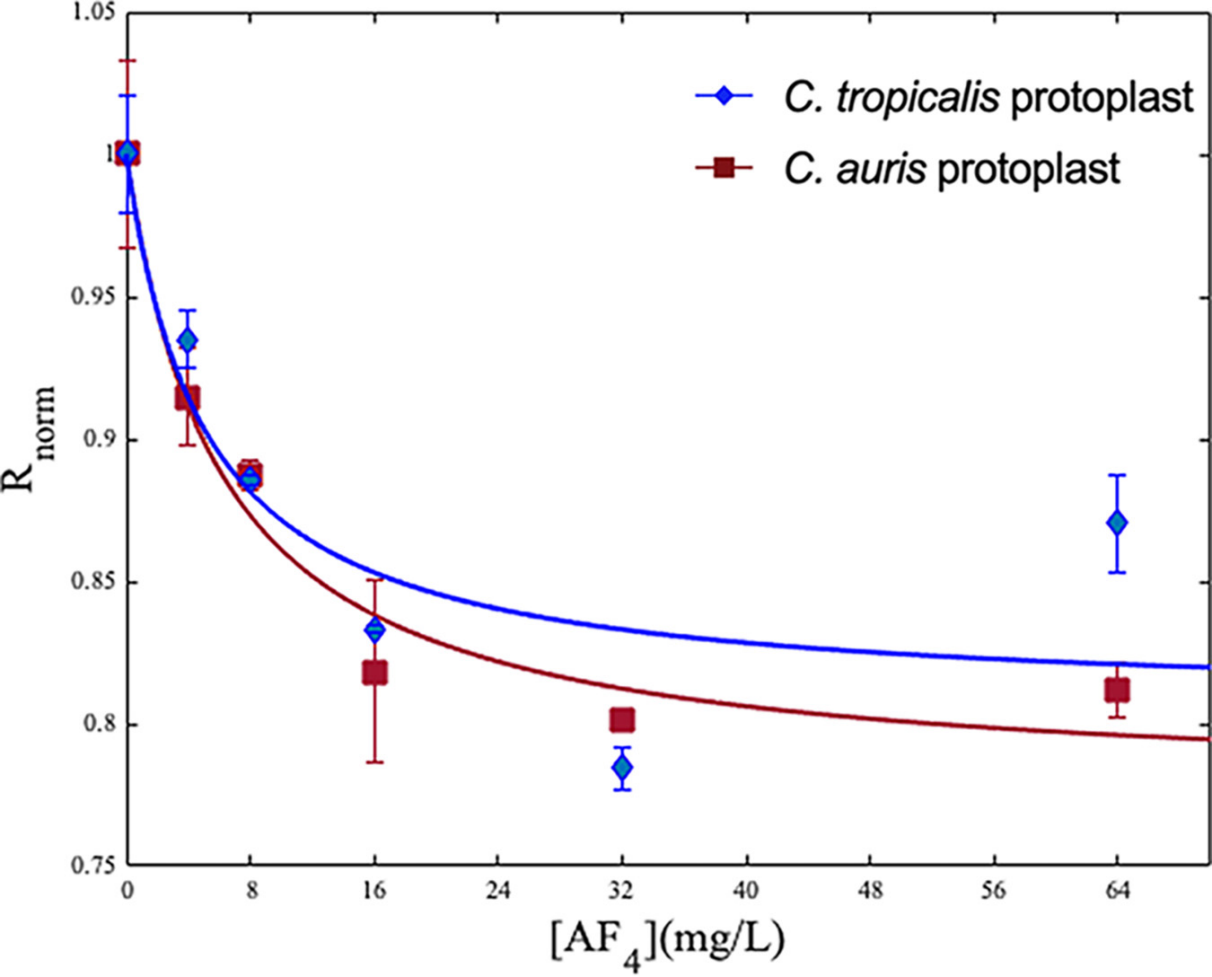
Effect of AF_4_ on membrane dipole potential. Interaction of AF_4_ with *Candida* protoplasts labeled with membrane potential sensitive probe di-8-ANEPPS [4-(2-[6-(dioctylamino)-2-naphthalenyl] ethenyl)-1-(3-sulfopropyl) pyridinium inner salt]. *R*_norm_ values were obtained by normalizing *R* values with the ratio obtained for untreated protoplasts. The values of the normalized excitation ratio, *R*_norm_ (*R*/*R*_0_), were used to fit the experimental data to equation 1 (in Materials and Methods) by nonlinear regression using MATLAB R2022b. Data are presented as means ± SD.

The interaction of lipopeptide with the plasma membrane of *Candida* cells was assessed by changes in the membrane potential of protoplasts labeled with 4-(2-[6-(dioctylamino)-2-naphthalenyl] ethenyl)-1-(3-sulfopropyl) pyridinium inner salt (di-8-ANEPPS) (Fig. 6). The variation in ratios of fluorescence intensities at 455 and 525 nm was recorded after treatment with a range of concentrations of AF_4_ and a decrease in membrane dipole potential was observed. This decrease is indicative of the interaction between the membrane and lipopeptide. The apparent dissociation constants (*K_d_*) were found to be 5.046 ± 4.7 mg/L for C. tropicalis protoplasts and 6.095 ± 2.05 mg/L for C. auris protoplasts, respectively.

### Membrane integrity studies using FC analysis.

**FIG 7:**
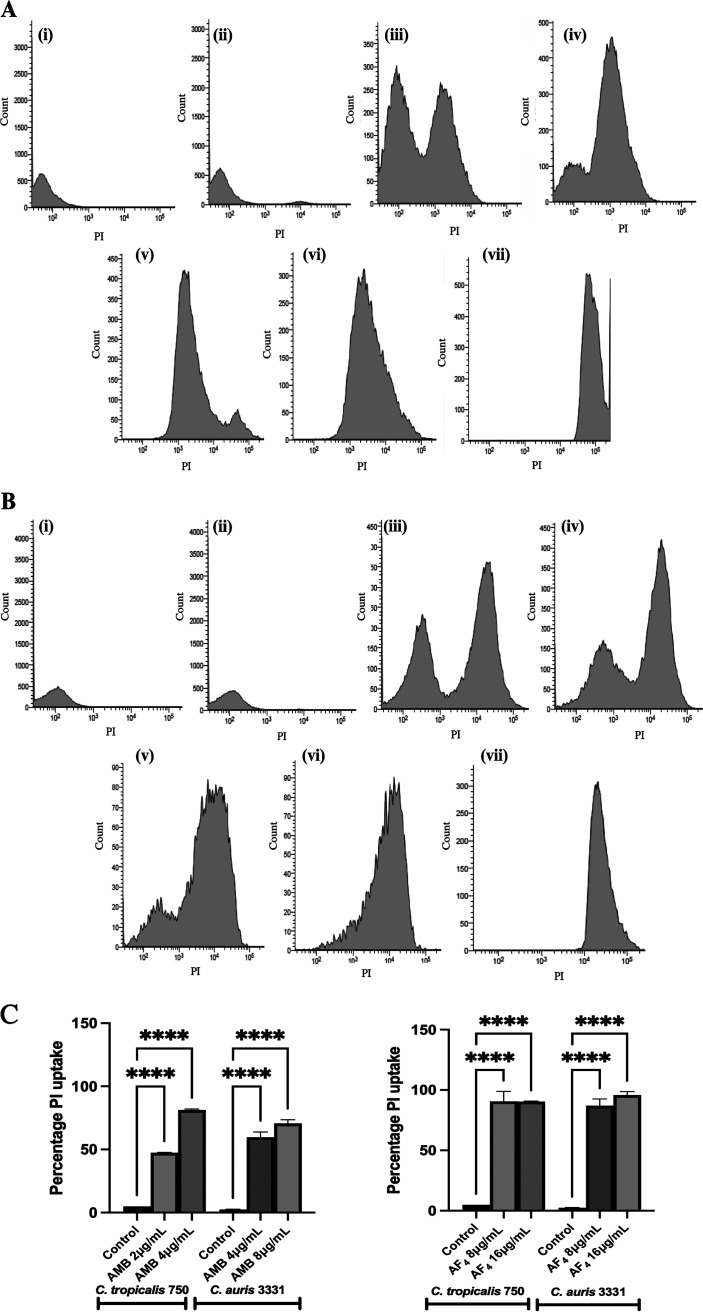
Flow cytometric (FC) analysis of membrane stability. (A) Histograms of C. tropicalis ATCC 750 cells stained with PI, analyzed using a 488-nm laser with a PI filter (586 nm). (i) Unstained cells, (ii) cells without antifungal drug treatment, and cells treated with (iii) 2 mg/L AMB, (iv) 4 mg/L AMB, (v) 8 mg/L AF_4_, (vi) 16 mg/L AF_4_, or (viii) 70% ethanol (30,000 events). (B) Histograms of C. auris IL-3331 cells stained with PI, analyzed using a 488-nm laser with a PI filter (586 nm). (i) Unstained cells, (ii) cells without antifungal drug treatment, and cells treated with (iii) 4 mg/L AMB, (iv) 8 mg/L AMB, (v) 8 mg/L AF_4_, (vi) 16 mg/L AF_4_, or (vii) 70% ethanol (AMB: 30,000 events; AF_4_:10,000 events). (C) Analysis of the percentage of cells positively stained by PI after (i) treatment with AMB for 3 h or (ii) treatment with AF_4_ for 18 h. For both experiments, the percentage of PI uptake shown below is averaged across two experiments. *P < *0.0001 compared with the control using one-way ANOVA followed by Tukey’s test. All event FC dot plots are shown in Fig. S5 in the supplemental material.

Flow cytometry-based detection of altered permeability of the cell membrane was performed using the vital stain PI (20) following treatment with lipopeptide AF_4_ and AMB. When C. tropicalis and C. auris cells were treated with the antifungal variant AF_4_, cell membrane damage was inferred from the increased fluorescence resulting from the uptake of PI. In comparison, untreated cells showed absence or negligible PI fluorescence. The increased fluorescence in cells treated with AMB, AF_4_, and 70% ethanol is seen as a clear shift of peak along the *x* axis (Fig. 7A and B, Fig 7C panel iii, and Table 2). In all these cases, ethanol-treated yeast cells exhibited a high permeability to PI (>99% cells stained) and a 99.9% reduction in CFU as recorded from the plate counts (not shown for ethanol). Fig. 7A and B are representative histograms from experiments in which C. tropicalis and C. auris cells were stained with PI following a 3-h treatment with AMB and an 18-h treatment with AF_4_ (8 and 16 mg/L). All event FC dot plots are shown in Fig. S3A and B.

**TABLE 2:** Correlation of PI uptake percentage from flow cytometry and plate counts for C. tropicalis ATCC 750 and C. auris IL-3331^*a*^

Strain and sample	FC PI uptake (%)	Plate count	
		Log reduction	Reduction (%)
Candida tropicalis ATCC 750			
Unstained	0.01	-^*b*^	-
Untreated	4.9	-	-
AMB (2 mg/L)	47.4	4.47	99.99
AMB (4 mg/L)	81.3	5.35	99.99
AF_4_ (8 mg/L)	90.5	1.05	91.16
AF_4_ (16 mg/L)	90.4	1.27	94.63
C. auris IL-3331			
Unstained	0.05	-	-
Untreated	1.7	-	-
AMB (4 mg/L)	49.9	4.61	99.99
AMB (8 mg/L)	60.3	2.44	99.64
AF_4_ (8 mg/L)	86.6	3.03	99.90
AF_4_ (16 mg/L)	97.9	4.08	99.99

a FC, flow cytometry; PI, propidium iodide; AMB, amphotericin B.

b -, not applicable.

Comparison of PI uptake percentages and reductions in CFU/mL across AF_4_ treatments showed good congruence. The percentage reduction observed in plate counts was highly comparable to the percentage of PI uptake recorded in flow cytometry, indicating an excellent correlation between these two, as summarized in Table 2. At 8 mg/L, the AF_4_-treated C. tropicalis showed 90.5% PI-positive cells (Table 2, Fig. 7C) and 90.16 CFU reduction; this was similar to that observed in AF_4_-treated C. auris cells, where 86.6% of cells were PI-positive (Table 2, Fig. 7C) and a CFU reduction of 99.90% was recorded.

Morphological changes due to antifungal treatment were observed by flow cytometric analysis of the forward scatter (FSC) (cell size, *x* axis values) and side scatter (SSC) (cell complexity/granularity, *y* axis) values. It was observed that upon treatment with AF_4_, cell size and granularity/complexity increased by an average of 17.9% in C. tropicalis and an average of 45% in C. auris across the two concentrations tested (Fig. S6A and B). Cells treated with AMB showed a decrease in size and an increase in complexity for both strains used, and those treated with ethanol showed a drastic decrease in size (Fig. S6A and B).

### Evaluation of ROS production.

**FIG 8:**
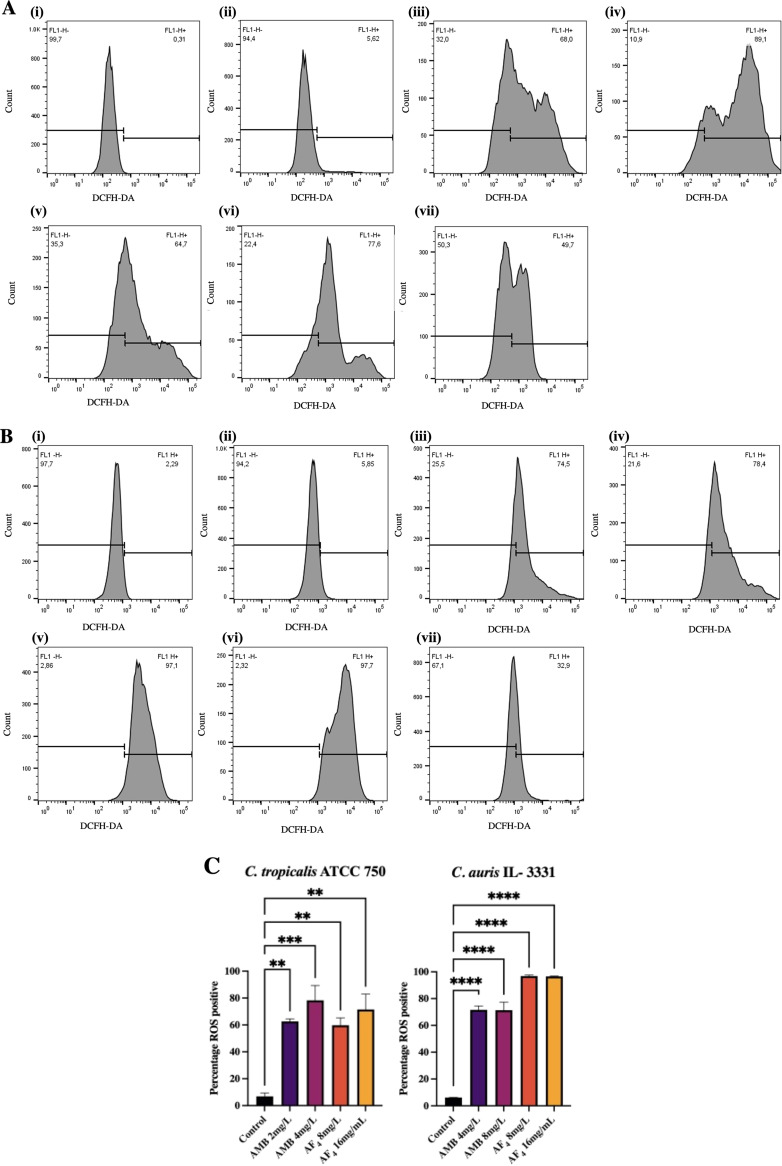
Flow cytometric analysis of reactive oxygen species (ROS) generation upon treatment. (A) FC histograms of C. tropicalis ATCC 750 cells stained with DCFH-DA (2′,7′-dichlorodihydrofluorescein diacetate) analyzed using an FITC (fluorescein isothiocyanate) filter. Panels showing percentage of DCFH-DA-positive cells (FL1), indicating the ROS generated by (i) unstained cells, (ii) untreated cells, and cells treated with (iii) 2 mg/L AMB, (iv) 4 mg/L AMB, (v) 8 mg/L AF_4_, (vi) 16 mg/L AF_4_, or (vii) 10 mM H_2_O_2_. (B) FC histograms showing traces of C. auris IL-3331 cells stained with DCFH-DA analyzed using an FITC filter. Percentage of DCFH-DA positive cells (FL1) indicating the ROS generated by (i) unstained cells, (ii) untreated cells, and cells treated with (iii) 4 mg/L AMB, (iv) 8 mg/L AMB, (v) 8 mg/L AF_4_, (vi) 16 mg/L AF_4_, or (vii) 10 mM H_2_O_2_ (C) Percentage of ROS-positive cells in C. tropicalis and C. auris across treatments. Results represent mean ± SD and *P* values (*P = *0.0014 for C. tropicalis and *P < *0.0001 for C. auris) denote statistically significant differences compared to control assessed by one-way ANOVA. A total of 20,000 events were analyzed.

Reactive oxygen species (ROS) generation is a key marker of antifungal activity and of cells undergoing early apoptosis (21). Using the fluorescent dye 2′,7′-dichlorodihydrofluorescein diacetate (DCFH-DA), we estimated ROS accumulation in treated C. tropicalis and C. auris cells. The intracellular ROS produced upon treatment causes oxidation of DCFH-DA to 2’,7’-dichlorofluorescein (DCF), resulting in enhanced fluorescence. As shown in Fig. 8A and B, the AF_4_-treated yeasts displayed elevated levels of ROS generation inferred from the increased fluorescence. C. tropicalis showed 64% and 77% ROS-positive cells at 8 and 16 mg/L, respectively. AF_4_-treated C. auris cells also showed a 97% increase in ROS-positive cells at both 8 and 16 mg/L (Fig. 8C). The ROS generated in AMB-treated cells of both organisms also produced a significant increase in fluorescence.

### Modifications to mitochondrial membrane potential.

**FIG 9:**
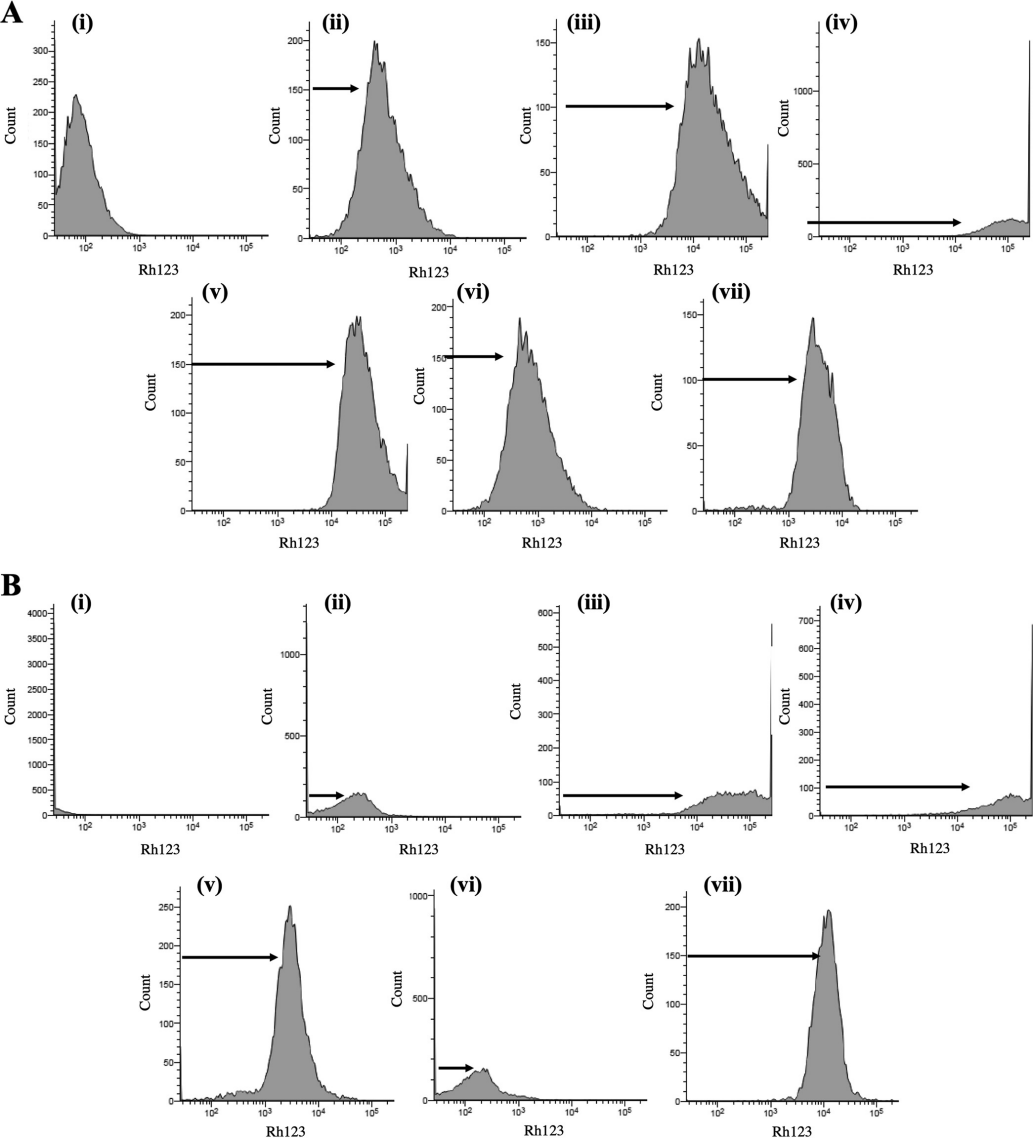
Assessment of mitochondrial membrane potential and dysfunction using Rhodamine 123 (Rh123). (A) FC histograms of C. tropicalis ATCC 750 cells stained with Rh123 analyzed using an FITC filter. Results are shown for (i) unstained cells, (ii) untreated cells, and cells treated with (iii) 8 mg/L AF_4_, (iv) 16 mg/L AF_4_, (v) 70% ethanol, (vi) sodium azide (40 mM), or (vii) 121°C heat-treatment. (B) FC histograms of C. auris IL-3331 cells stained with Rh123 analyzed using an FITC filter. Results are shown for (i) unstained cells, (ii) untreated cells, and cells treated with (iii) 8 mg/L AF_4_, (iv) 16 mg/L AF_4_, (v) 70% ethanol, (vi) sodium azide (40 mM), or (vii) 121°C heat- treatment. A total of 10,000 events were analyzed in both experiments.

The effect of antifungal treatment on mitochondrial membrane potential is inferred from the accumulation of rhodamine 123 (Rh123) within the mitochondrial membrane. After 18 h exposure to antifungal AF_4_ (8 and 16 mg/L), increased fluorescence of Rh123 was seen as a shift of the histogram along the *x* axis to the right (as shown in Fig. 9A and B, panels iii and iv); a similar shift was observed in ethanol-treated (panel v) and heat-treated (panel vii) cells. This increased fluorescence can be attributed to non-localized accumulation of Rh123 resulting from cell death (22). Sodium azide is a known inhibitor of oxidative phosphorylation and, at a concentration of 40 mM, showed a marginal shift to the left, indicating a decrease in fluorescence (as shown in Fig. 9A and B, panel vi).

The shift of peaks indicating fluorescence along the *x* axis was also used to observe the extent of cell death using Rh123 as a live/dead probe (Fig. S7A and B). The dot (pseudocolor) plots of SSC height versus Rh123 height of treated samples were used to infer cell death by comparing them with the profile obtained for untreated cells. Of the lipopeptide-treated cells at 8 and 16 mg/L, a significant number lay beyond gate P7 compared to that in the control samples. Cells lying within gate P7 (set according to control samples) were considered live cells and those in gate P8 were considered to have significantly damaged membranes and were therefore recorded as dead cells (Fig. S7A and B).

### Nuclear fragmentation and DNA damage.

**FIG 10:**
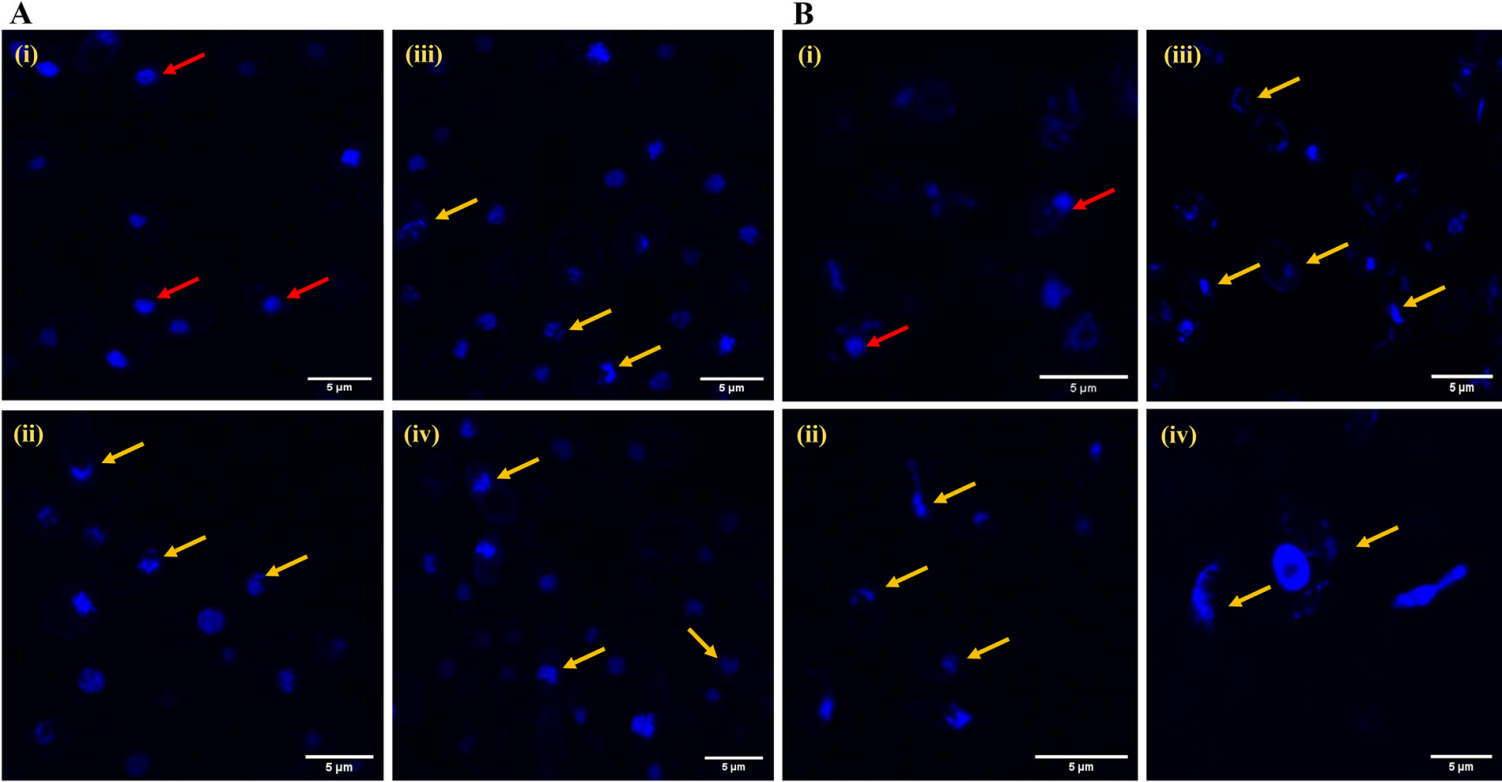
Visualizing DNA damage by DAPI (4′,6-diamidino-2-phenylindole) staining. (A) C. tropicalis ATCC 750 cells, showing (i) untreated cells and cells treated with (ii) 2, (iii) 4, or (iv) 8 mg/L f AF_4_ lipopeptide. (B) C. auris IL-3331 cells with DAPI, showing (i) untreated cells and cells treated with (ii) 2, (iii) 4, or (iv) 8 mg/L AF_4_ lipopeptide. Red arrows indicate intact nuclei; yellow arrows indicate increased fluorescence and distorted nuclear morphology suggesting nuclear fragmentation and DNA damage. Scale bar = 5 μm.

DNA damage is considered a probable indicator of late apoptosis (21, 23). 4’,6-diamidino-2-phenylindole (DAPI), a membrane-permeable dye, shows fluorescence upon binding to nucleic acids, helping to visualize nuclear morphology and the extent of DNA damage, nuclear fragmentation, or condensation in response to antifungal treatments. Cells treated with AF_4_ at different concentrations showed increased fluorescence with damaged abnormal nuclei, including tube-like, teardrop-shaped, or distorted nuclei, as shown in Fig. 10A and B, indicated by yellow arrows. In comparison, untreated cells, indicated by red arrows in both panels, showed spherical nuclei with normal morphologies and fluorescence.

## DISCUSSION

Current antifungal therapies are limited to combinations or increased dosages of pre-existing drugs, while pushing for the development of novel antifungals with broad spectrum, reduced adverse reactions, and minimal cytotoxicity at fungicidal concentrations. In our previous investigations (13, 14), we described the broad-spectrum antifungal activity of a set of five antifungal lipopeptides variants isolated from B. subtilis. These antifungal lipopeptide variants demonstrated potent antifungal activity against many isolates of C. albicans, *Candida* non-*albicans*, Cryptococcus species, and filamentous fungi (13, 14). These antifungal variants have differing antifungal potencies, with the AF_4_ variant being the most potent, warranting further investigation into its effects on *Candida* non-*albicans* species.

The low MIC/MFC values of AF_4_ obtained from antifungal susceptibility testing encouraged further investigation of its antifungal efficacy and cytotoxicity. We obtained promising results from the time-killing assay, showing that AF_4_ has significant antifungal activity against C. tropicalis and C. auris. The reduction in cell numbers was observed to begin at 6 h, with continued reduction in CFU/mL until 24 h at both 16 and 32 mg/L. The antifungal activity of AF_4_ against C. tropicalis is evidenced by CFU/mL log reductions of 1.212 ± 0.0156 CFU/mL at 16 mg/L and 1.337 ± 0.125 CFU/mL at 32 mg/L. Similarly, the log reduction in CFU/mL of C. auris treated with 16 mg/L AF_4_ was 1.428 ± 0.0082 CFU/mL, and with 32 mg/L it was 1.809 ± 0.269 CFU/mL. These results suggest that the activity of AF_4_ manifests from 12 h onwards and shows significant fungicidal activity until 24 h (Fig. 1). In AMB-treated cells, the fungicidal activity was found to manifest after 3 h and persisted until 48 h, indicating the potent fungicidal activity of AMB. The reduction in cell numbers in all the experiments was persistent, suggesting that AF_4_ is adequately fungistatic against organisms in *in vitro* pharmacodynamics assays.

The MTT assay showed that AF_4_ exhibits low to moderate cytotoxicity at its MIC/MFC values against the three cell lines tested (Fig. 2) (24). Cell viability was found to be >50% at 8 mg/L, which is higher than the MIC values observed for each of the target *Candida* strains (used in the present study), with RAW264.7 and NIH 3T3 cells showing ~75% viability. Significant cytotoxicity has been observed only at high concentrations of 16 mg/L and greater, which is twice the MFC value (8 mg/L) and at which strong fungicidal activity has been observed against all target pathogens (Table S1). AMB showed significant cytotoxicity when tested at 8 mg/L against NIH 3T3 and RAW264.7 and low cytotoxicity for Vero cell lines. The IC_50_ values obtained for all three cell lines were found to be more than the MFC (8 mg/L), and a significant difference was observed between the IC_50_s of AMB and AF_4_, indicating that AF_4_ is biocompatible and shows reduced toxicity to cultured cells, encouraging consideration of AF_4_ as an antifungal lead molecule.

Because the cell membrane is most often the primary potential target, changes in cell morphology due to lipopeptide action were visualized using microscopy. Scanning electron micrographs post-treatment with AF_4_ showed membrane perturbations and inward collapse of the cell membrane. Compared to untreated cells, cells treated with AF_4_ and AMB showed two types of damage: inward curving of the cell membrane and surface deformities (Fig. 3). This appearance of the damaged cells suggests that the lipopeptide follows the toroidal pore-forming mode of action in which the peptide is adsorbed on the membrane surface, causing progressive thinning and bending of the bilayer which results in membrane damage (25).

Confocal microscopy imaging was used to further visualize the damage AF_4_ causes to the cell membrane and to determine whether damage to the membrane caused permeabilization. Images of C. tropicalis and C. auris treated with antifungal AF_4_ and AMB exhibited orange-yellow fluorescence (Fig. 4A and C, panels ii and iii) indicating the uptake of AO, the membrane-permeable live cell stain, and PI membrane-impermeable dye, which selectively stains membrane-compromised cells due to membrane permeabilization. In comparison, untreated cells showed only green fluorescence, attributed to the uptake of only AO (Fig. 4A and C, panel i). This is supported by images obtained using the membrane-permeable two-color stain FUN-1, which showed that cells treated with AF_4_ and AMB significantly lose their metabolic activity, appearing diffuse yellow-green (Fig. 4B and D, panels ii and iii), whereas peptide-untreated cells that are metabolically viable are diffuse green and capable of forming the characteristic CIVS (Fig. 4B and D, panel i) (18, 26–29). It could be speculated that the lack of metabolic activity is associated with membrane damage caused by AF_4_ or the interaction of AF_4_ with other cell organelles, resulting in cell death.

Because membrane-impermeable dyes were able to cross the membrane, further studies on fungal membrane dynamics were performed to understand the effect of AF_4_ on the membrane. Ergosterol is a major sterol of the fungal plasma membrane and regulates membrane stability and fluidity (30). The mode of action of standard antifungal AMB is based on binding to the ergosterol present in fungal cell membranes and pore formation leading to ion leakage and cell death (31). The ergosterol binding assay showed that AF_4_ can bind to the ergosterol present on cell membranes. The increased MICs observed in the presence of exogenous ergosterol are indicative of the complexation of lipopeptide and ergosterol of the cell membrane and the subsequent effect of AF_4_ on cell membrane dynamics (19, 32, 33). Similar binding and increased MIC values were also observed with AMB-treated C. tropicalis and C. auris cells. These results may suggest that AF_4_ binds ergosterol and destabilizes the membrane. DPH assays provided further insight into the membrane dynamics upon treatment with AF_4_. DPH is a fluorescent molecule that is incorporated into membrane phospholipids without disturbing the lipid bilayer. Cells treated with AF_4_ and AMB showed a significant decrease in DPH fluorescence (Fig. 5) in comparison to untreated cells, indicating that the cell membrane was disrupted and damaged, impeding DPH binding to the membrane (34–36). Additionally, the determination of ergosterol content discussed in the supplemental materials showed that the antifungal activity of AF_4_ (Fig. S4) caused a reduced proliferation of cells, evidenced by the lower recovery of ergosterol in treated cells compared to that in untreated cells.

To observe changes to the dipole potential of the plasma membrane upon treatment with AF_4_, we used the membrane probe di-8-ANEPPS. Di-8-ANEPPS, a potentiometric probe, is incorporated into the outer leaflet of the plasma membrane and is used to observe membrane depolarization. Cells labeled with di-8-ANEPPS are expected to show a decrease in dipole potential when the peptides bind by adsorption or insertion (37) into the membrane. Here, a decrease in the normalized ratio of fluorescence intensities in lipopeptide-treated samples was observed with increasing concentrations of lipopeptide AF_4_ (Fig. 6). This concentration-dependent decrease in the intensity ratio corresponds to a reduction in membrane potentials. These results showed that the higher the initial dipole potential, the greater its decrease, driven by the interaction of the lipopeptide with the cell membrane. A similar observation and inference was made in a peptide-biomembrane interaction study using the di-8-ANEPPS-based membrane dipole potential (38, 39). The *K_d_* values obtained also indicate considerable affinity of AF_4_ with the plasma membrane.

Considering the results of the ergosterol binding assay, DPH assay, membrane dipole potential analysis, and ergosterol extraction assay, AF_4_ appears to impact the fungal cell membrane by binding the membrane ergosterol and disrupting the plasma membrane, leading to loss of cell viability. A study on the interactions of lipopeptide mycosubtilin and ergosterol-containing artificial monolayers by Nasir and Besson (40) proposes that at the cell level, lipopeptide interaction may occur in two steps: first, the ergosterol molecules present in the outer leaflet of the plasma membrane interact with the peptide part; second, the lipid moiety of mycosubtilin interacts with the aliphatic chain of ergosterol. In another study by Zhang et al. (41), it was observed from the fluorescence polarization results of *Rhizopus solani* treated with the lipopeptide, bacillomycin L, that bacillomycin L and AMB both altered fungal membrane fluidity due to their interactions with membrane ergosterol. It was agreed that iturin lipopeptides interact strongly with sterols by forming a complex with membrane sterols and phospholipids (41). Similarly, an AF_4_/phospholipid/ergosterol complex formation may be triggering the antifungal activity. It could also be postulated that lipopeptide AF_4_ follows binding strategies similar to those described above, enabling subsequent membrane perturbation which enhances membrane permeability. Flow cytometry using PI was performed to further investigate the permeabilization of membranes.

Flow cytometry analysis with PI proved to be a promising method to determine the susceptibility of *Candida* cells to the antifungal lipopeptide AF_4_. The percentage of cells that were able to incorporate PI into nucleic acid describes the extent of membrane damage caused by AF_4_. PI was taken up sufficiently by yeast cells whose membranes were compromised when treated with 8 mg/L of AF_4_, which was reflected in the increased fluorescence (42) (Fig. 7A and B). The PI uptake results (Table 2) showed a good correlation with the colony counts and percentage reduction of CFU/mL calculated after treatment with 8 mg/L, suggesting that the damage to membranes caused by AF_4_ at the MFC values is highly perturbing, resulting in cell death (36). However, PI uptake in AMB-treated cells was not reflective of the extent of reduction in CFU/mL, probably due to the interference with PI uptake caused by the mode of action of polyene AMB (20, 42–45).

These results indicate that membrane disruption may be a key mode of action for the antifungal lipopeptide AF_4_, which is also evidenced by confocal microscopy images in which membrane permeabilization results in the entry of membrane-impermeant fluorescent dyes. The increased FSC (Fig. S6A and B) observed in flow cytometry suggests that AF_4_ may create surface pores without affecting cell size due to the lipopeptide’s interaction with membrane sterols, as discussed above (46). The increased SSC, indicative of increased cell complexity/granularity, may be correlated with the dimpled and deformed surfaces revealed in SEM images.

The lipopeptide’s ability to cause membrane disruption without a change in cell size led us to investigate its effects within the cell. ROS generation is an early apoptosis marker, an indicator of cellular dysfunction and death (21, 23). Recent research indicates that oxidative damage triggered by intracellular ROS buildup is a universal phenomenon in the fungicidal effect exerted by AMB against pathogenic yeasts (47–49). The AMB- and AF_4_-treated yeasts showed increased fluorescence (Fig. 8A and B), indicating the formation of ROS intracellularly. Endogenous ROS accumulation may then trigger a series of intracellular events and oxidative damage to DNA, proteins, and lipids (50), leading to fungal cell death (51–53). Our results (Fig. 8A and B) suggest that AF_4_-induced intracellular ROS generation is one of the likely mechanisms for the candidacidal activity of AF_4_. Flow cytometric analysis of mitochondrial membrane potential revealed that an increase in fluorescence occurred upon treatment with AF_4_ (Fig. 9A and B). The AF_4_ potentially causes a loss of mitochondrial membrane integrity, and an absence of fluorescence quenching is evident (as shown in Fig. 9A and B) as the Rh123 fails to localize into the mitochondrial membrane. This increased fluorescence could also be a result of the interaction of Rh123 with damaged nucleic acids, proteins, and other cell constituents due to cell death (Fig. S7A and B) (22). A similar enhanced fluorescence was recorded in heat-treated and ethanol-treated cells. It could be postulated that the cell membrane damage resulted in cellular stress response-mediated ROS production (54, 55) The ROS thus produced may lead to mitochondria dysfunction. Alternatively, reports have also suggested that increased ROS production could also be attributed to pore formation (56) on the mitochondrial membrane and the release of molecules, causing oxidative damage and cellular stress.

Nuclear fragmentation and DNA damage are indicators of cells undergoing late apoptosis (21, 23). It has also been found that certain echinocandins inflict apoptosis only at sub-MICs and MICs and not beyond the MICs (21). Significant changes in nucleus morphology upon treatment with AF_4_ were observed at 2 and 4 mg/L (Fig. 10A and B). The DNA damage may be due to cellular ROS generation. These observations also corroborate previous findings, as the lipopeptide above the MIC values rarely inflicted extensive nuclear disintegrations, as shown in Fig. 10A and B.

Taken together, our results indicate a multipronged antifungal action of the lipopeptide AF_4_ (Fig. S8). AF_4_ primarily targets the fungal cell membrane, causing severe membrane perturbation and permeabilization by potentially binding the ergosterol present in the plasma membrane. The ergosterol binding and membrane dipole potential experiments indicate that the binding of the lipopeptide to the cell membrane, either by adsorption or insertion, causes changes in the membrane structural properties (37). This alteration to the physical or chemical properties of the membrane may induce membrane stress, which in turn results in ROS generation and accumulation within the cells (55). The investigated lipopeptide thus exerted its antifungal activity by targeting the plasma membrane, leading to membrane depolarization, membrane permeabilization, accumulation of endogenous ROS leading to mitochondrial dysfunction, and nuclear DNA damage.

This lipopeptide has demonstrated highly promising antifungal activity against *Candida* non-*albicans* strains, comparable to that of standard antifungals. These observations warrant further investigations to gain deeper mechanistic insights into its antifungal mode of action. They also encourage investigations into the use of the AF_4_ lead molecule as a potential alternative therapeutic (for topical application) and a broad-spectrum antifungal candidate.

## MATERIALS AND METHODS

### Microorganisms.

All the isolates (clinical) and strains of C. tropicalis and C. auris that were used in this study were obtained from the National Culture Collection of Pathogenic Fungi (NCCPF), Post Graduate Institute of Medical Education and Research (PGIMER; Chandigarh, India) and maintained as 20% glycerol stock at −80°C.

### Extraction and purification of antifungal compounds.

Briefly, cell-free supernatant was prepared from B. subtilis RLID 12.1 followed by an optimized three-step purification process (14, 57). The active fraction AF_4_ was isolated as one of the five compounds as described in Ramya et al. (13). The semipreparative scale RP-HPLC system consists of an Agilent quaternary pump (Agilent Technologies, USA) and a variable wavelength detector equipped with a Phenomenex Luna C_18_ column (10 mm × 250 mm, 5 μm) (Phenomenex, USA) enabled the purification process (14). Out of the five fractions, the variant AF_4_ was chosen for the current study because it exhibited the highest and broadest spectrum of antifungal activity (13, 14).

### Antifungal susceptibility testing against *Candida* non-*albicans*.

The MICs and MFCs of the purified antifungal AF_4_ variant were tested against C. tropicalis ATCC 750, 5 clinical isolates of C. tropicalis, and 4 clinical isolates of C. auris according to CLSI guidelines (M27-A3) (58). C. albicans SC5314 and C. glabrata ATCC 2001 were used as the reference yeast strains, and the standard antifungal amphotericin B (AMB) (HiMedia, India) was used as a positive control.

### *In vitro* pharmacodynamics of AF_4_ tested against *Candida* non-*albicans* strains.

The HPLC-purified AF_4_ fractions were tested for time-dependent activity against C. tropicalis ATCC 750 and C. auris IL-3331 at concentrations of 16 and 32 mg/L as described by Ramya et al. (13) and Clancy et al. (59). AMB, at 2× and 4× MIC, was used as a positive control against each strain/isolate tested. Briefly, the cell suspension was added to drug-free RPMI 1640 medium (HiMedia, India) and RPMI 1640 with the drug. The suspensions were incubated at 37°C for 24 h and at regular time intervals, an aliquot was removed, serially diluted, plated, and incubated for 24 h. To determine fungicidal activity, the CFU count at each time point was used to calculate percentage and log-reduction values in comparison to cells growing in drug-free RPMI. The experiments were conducted in duplicate.

### Biocompatibility testing using MTT assay.

The three cell lines used in this study, murine fibroblast NIH 3T3, murine monocyte/macrophage RAW 264.7, and Vero were obtained from the National Centre for Cell Science (NCCS), Pune, India, to assess the viability of cells in the presence of the antifungal lipopeptide AF_4_ and AMB. Cell viability testing by MTT assay (HiMedia, India) was used to determine the percentage of metabolically active cells, which indicated the percentage of viability and biocompatibility of drug-treated cells. Approximately 5,000 cells were seeded in 96-well plates into a growth medium supplemented with 10% fetal bovine serum (FBS) (HiMedia, India) and 1% antibiotic (penicillin [5,000 U] and streptomycin [5 mg/mL]; HiMedia, India) solution. After 24 h of incubation at 37°C in a 5% CO_2_-supplemented incubator, spent medium was aspirated and fresh medium with AF_4_ and AMB at a range of concentrations was added. After 24 h, 2 mg/mL MTT was added to the media in each well and incubated for 3 h, and formazan crystals formed were visualized. Formazan crystals were solubilized by incubating for 15 min with dimethyl sulfoxide (DMSO). Absorbance at 570 nm was read using a Multiskan Go plate reader (Thermo Fisher Scientific, USA) and 50% inhibitory concentration (IC_50_) values were calculated for each cell line (24, 60).

### Field-emission scanning electron microscopy.

Scanning electron microscopy (SEM) was used to visualize the effect of AF_4_ and AMB on C. tropicalis ATCC 750 and C. auris IL-3331 membranes. The cells were grown in drug-free medium, medium with the antifungal drug AF_4_ (8 mg/L) overnight, and medium with AMB (2× MIC) for 3 h at 37°C under shaking conditions. After incubation, cells were centrifuged at 10,000 rpm, washed with 1× phosphate-buffered saline (PBS) (HiMedia, India) and fixed onto coverslips using 2.5% glutaraldehyde (Sigma-Aldrich, USA) in 0.1 M cacodylate buffer (Sigma-Aldrich) for 3 h at 4°C. Post-fixation, the sample was washed with 0.1 M sodium cacodylate buffer and sterile distilled water to remove traces of fixative. After washing, 20 μL of 1% osmium tetroxide was added, and the sample was incubated for 1 h and dehydrated using a graded ethanol series (61, 62). The sample was then dried by critical point drying (Leica EM CPD 300, Leica Microsystems, Germany) and sputter-coated with 5 nm of gold (Leica EM ACE 200). The images were captured using a FESEM (field-emission scanning electron microscope) Quanta FEG 250 (Thermo Fisher Scientific, USA) instrument (CSI Facility, BITS Pilani Goa Campus) at ×20,000 magnification.

### Confocal laser scanning microscopy.

We performed a confocal laser scanning microscopy (CSLM) study using two different staining techniques—a combination of AO (HiMedia, India) and PI (Sigma-Aldrich, USA) and the two-color stain FUN-1 (Thermo Fisher Scientific, USA) to observe the mode of action of purified antifungal variant AF_4_. For both staining methods, an inoculum with ~5 × 10^6^ CFU/mL was prepared and incubated with the antifungal drug at a concentration of 8 mg/L for 18 h, respectively, at 37°C under shaking condition. Cells were incubated with AMB at 2 mg/L (C. tropicalis) and 4 mg/L (C. auris) for 3 h at 37°C under shaking condition, and cells grown without any drug treatment were used as controls. Cells were harvested at 10,000 rpm and AO was added to the resuspended cell pellet at a concentration of 20 μM and PI was added at 5 μg/mL (26, 27). The samples were imaged using an Olympus FV3000 microscope (Olympus Corporation, Japan) (CSI Facility, BITS Pilani Goa Campus) at different magnifications (26, 27). Stains were added individually and incubated in the dark for 15 min at 37°C each with an intermittent wash using 1× PBS to remove unbound stains.

The FUN-1 stain was prepared in glucose-HEPES (GH) buffer and added at a final concentration of 5 μM. Cells were incubated in the darkness for 30 min at 30°C. GH buffer solution was used to wash off unbound stains before imaging (18, 28, 63). The fluorescence filters used are detailed in the figure legends.

### Ergosterol binding assay.

To determine the binding of AF_4_ to the ergosterol present in fungal cell membranes, an ergosterol binding assay was performed as described by Escalante et al. (32) with minor modifications. The ergosterol was prepared by dissolving in DMSO and Tween 80. The emulsion was heated and homogenized to improve solubility. The prepared emulsion was then added to RPMI 1640 at 100, 200, and 400 μg/mL. The MICs of AF_4_ and AMB against C. tropicalis ATCC 750 and C. auris IL-3331 were determined according to CLSI guidelines with medium supplemented with ergosterol and medium without ergosterol. Briefly, AF_4_ (64 mg/L) was double-diluted in wells of a 96-well microtiter plate with 100, 200, and 400 μg/mL of ergosterol in RPMI or in RPMI without ergosterol. As a control, the same procedure was performed for AMB (16 mg/L) in separate microtiter plates. Approximately 10^3^ cells of yeast cell suspensions were added to each well and the plates were incubated at 37°C for 48 h. The changes in MICs in the presence and absence of exogenous ergosterol were recorded to infer the ergosterol binding capacity of the drugs used in the study.

### Measurement of plasma membrane fluorescence.

Changes to the membrane dynamics were assessed by labeling fungal cell membranes with DPH (Sigma-Aldrich, USA). The fluorescence emitted by DPH upon its intercalation with the fungal cell membrane is used to determine changes to the membrane lipid bilayer across treatments. Cells were treated with AF_4_ (8 mg/L) and AMB at 1× MIC for 18 and 3 h, respectively, at 37°C under shaking conditions. Cells were then fixed with 0.37% formaldehyde and incubated for 30 min at 28°C. Cells were then washed with PBS and flash-frozen using liquid nitrogen. Cells were subsequently thawed, resuspended in 1× PBS, labeled with 0.6 mM DPH, and incubated for 45 min at 28°C. Post incubation, cells were washed with PBS and homogenized by sonication on ice (34–36). Cells were then centrifuged, and the fluorescence intensity of the supernatant was measured using a spectrofluorometer (JASCO FP-8500, Japan) at 350 nm excitation and 425 nm emission.

### Measurement of the membrane dipole potential.

The change in dipole potential of *Candida* cells was determined using fluorescence intensities of the styryl dye di-8-ANEPPS (Sigma-Aldrich, Germany) as described by de Aguiar et al. (64). *Candida* cells were harvested and adjusted to 10^6^ CFU/mL. Protoplasts were prepared by treating *Candida* cells with lyticase enzyme (250U/mL) (HiMedia, India) for 70 min (65). A protoplast suspension of 10^5^ CFU/mL was prepared and incubated for 1 h at 25°C in the darkness with 100 μM di-8-ANEPPS in HEPES buffer with 0.1% Pluronic F-127 (Sigma-Aldrich, USA) with gentle stirring. Post-incubation, 10^4^ CFU/mL of the same suspension was incubated with 10 μM di-8-ANEPPS for 1 h at 25°C in the darkness with stirring. AF_4_ was added to this suspension at 4, 8, 16, 32, and 64 mg/L and incubated for 90 min. As controls, cell suspensions without fluorescent probe or lipopeptide treatments were used. The ratio of intensities was obtained using excitation wavelengths of 455 and 525 nm and an emission wavelength of 670 nm, with excitation and emission slits set at 5 nm and 10 nm, respectively, using a spectrofluorometer (JASCO FP-8500, Japan). The *K_d_* values were determined using the equation shown below by fitting the experimental data using MATLAB R2022b:

(1) $$R/R_{0}=1+\frac{\frac{R_{\text{min}}}{R_{0\;}}\times \left[{\text{AF}}_{4}\right]}{K_{d}+[{\text{AF}}_{4}]}$$

where *R* is the ratio of intensities at 455 nm and 525 nm at different lipopeptide concentrations and *K_d_* is the apparent dissociation constant.

### Propidium iodide (PI) uptake assay.

To study the effect of AF_4_ on membrane integrity, the antifungal lipopeptide AF_4_ (8 and 16 mg/L) and AMB (2× and 4× MIC) were added to the cell suspension (5 × 10^6^ CFU/mL) in RPMI 1640 and grown for 18 and 3 h, respectively, at 37°C under shaking conditions. Viable cells and 70% ethanol-treated cells were used as negative and positive controls for PI uptake, respectively. Post-incubation, cells were harvested at 10,000 rpm, washed, and stained with PI (7.5 μg/mL) for 20 min. Cell suspensions were analyzed using a FACScan flow cytometer (Becton, Dickinson, and Co., FACS Melody, USA) using a 488-nm laser line and a 586-nm filter for PI. For sample analysis, forward scatter, side scatter, and the percentage of PI-stained cells were collected. The data were processed using FlowJo version 10.8.1 software. In each experiment, untreated and unstained cells were sampled and analyzed first (20, 36, 42, 66).

### CFU assay.

Aliquots of yeast cultures were removed before the PI staining, serially diluted in sterile 1× PBS, and plated in duplicate on Sabouraud dextrose agar plates (43). The plates were incubated for 24 h at 37°C, colonies were counted, and results were expressed as the percentage of reduction in CFU/mL compared to the growth in the untreated sample.

### Determination of reactive oxygen species production.

Intracellular ROS levels were measured post-treatment using DCFH-DA (Sigma-Aldrich, USA) by flow cytometry. Cells at ~5 × 10^6^ CFU/mL in RPMI 1640 were treated with antifungal lipopeptide AF_4_ (8 and 16 mg/L) for 18 h and with 2× and 4× MIC of AMB for 3 h at 37°C under shaking conditions. As a positive control, cells treated with 10 mM H_2_O_2_ for 1 h were used. After incubation, cells were centrifuged at 10,000 rpm, washed, and incubated in PBS with 10 μM DCFH-DA for 30 min in darkness at 37°C (35). The cell suspension was washed twice and analyzed using a BD FACScan flow cytometer and FlowJo version 10.8.1 software. The increase in the percentage of fluorescent cells in treated samples was compared with that of untreated samples.

### Mitochondrial membrane potential assessment.

To determine whether the antifungal AF_4_ affected the mitochondrial membrane potential (ΔΨm) of the cells, we analyzed the cells with Rh123 (Sigma-Aldrich, USA). Generally, changes to membrane potential are observed by a shift in the fluorescence of Rh123 in flow cytometry. Cell suspensions with ~5 × 10^6^ CFU/mL were treated with AF_4_ (8 and 16 mg/L) for 18 h in RPMI 1640 at 37°C under shaking conditions. Heat-treated (121°C, 15 min) cell suspension, sodium azide-treated (40 mM) samples, untreated cells, and unstained cells were analyzed as controls. After treatment and incubation, cells were harvested at 10,000 rpm and Rh123 was added at a concentration of 1 mg/L for 15 min at 35°C; cells were then washed twice and incubated at 35°C for an additional 30 min and analyzed immediately (67) using a BD FACScan flow cytometer and FlowJo version 10.8.1 software. The decrease in fluorescence peak intensity due to the sequestration of Rh123 in the mitochondrial membrane indicates a change in the ΔΨm (22). The peaks obtained for each sample were compared to determine the effect of AF_4_ on the ΔΨm. Additionally, live and dead cells were distinguished by observing the side scatter when they were stained with Rh123.

### Nuclear fragmentation and DNA damage.

Nucleic acid damage such as DNA fragmentation and condensation due to treatment with the antifungal lipopeptide AF_4_ was assessed using DAPI (HiMedia, India), a nucleic acid stain. Cell suspensions of C. tropicalis and C. auris were treated with antifungal lipopeptide at 2-, 4-, and 8-mg/L concentrations to observe dose-dependent effects on the nucleic acid content of the cells. Cells (~5 × 10^6^ CFU/mL) were incubated with AF_4_ for 18 h in RPMI 1640 at 37°C under shaking conditions. Untreated cells were used to observe the intact nuclei. Cells harvested at 10,000 rpm post-treatment were stained with 1 μg/mL DAPI for 20 min at 37°C (21, 23). Cells were imaged using an Olympus FV3000 microscope (CSI Facility, BITS Pilani Goa Campus).

### Statistical analysis.

Every experiment was conducted in duplicate, and results were represented as mean ± standard deviation. Results were analyzed on GraphPad Prism 9 software using the appropriate statistical tests for each experiment, as detailed in the figure legends.

### Data availability.

All data have been represented as results in the main and supplementary sections. Raw data will be made available upon request.

## References

[1] Aguirre UJ. 2002. Oral candidiasis. [Spanish] Rev Iberoam Micol 19:17–21. 12716225

[2] Sánchez-Vargas LO, Ortiz-López NG, Villar M, Moragues MD, Aguirre JM, Cashat-Cruz M, Lopez-Ribot JL, Gaitán-Cepeda LA, Quindós G. 2005. Point prevalence, microbiology and antifungal susceptibility patterns of oral *Candida* isolates colonizing or infecting Mexican HIV/AIDS patients and healthy persons. Rev Iberoam Micol 22:83–92. doi:10.1016/s1130-1406(05)70014-0. 16107165

[3] Manfredi M, Polonelli L, Aguirre-Urizar J, Carrozzo M, McCullough M. 2013. Urban legends series: oral candidosis. Oral Dis 19:245–261. doi:10.1111/odi.12013. 22998462

[4] Marcos-Arias C, Vicente JL, Sahand IH, Eguia A, De-Juan A, Madariaga L, Aguirre JM, Eraso E, Quindós G. 2009. Isolation of *Candida dubliniensis* in denture stomatitis. Arch Oral Biol 54:127–131. doi:10.1016/j.archoralbio.2008.09.005. 18950745

[5] Miranda-Cadena K, Marcos-Arias C, Mateo E, Aguirre JM, Quindós G, Eraso E. 2018. Prevalence and antifungal susceptibility profiles of *Candida glabrata*, *Candida parapsilosis* and their close-related species in oral candidiasis. Arch Oral Biol 95:100–107. doi:10.1016/j.archoralbio.2018.07.017. 30096698

[6] Chakrabarti A. 2015. *Candida glabrata* candidemia. Indian J Crit Care Med 19:138–139. doi:10.4103/0972-5229.152753. 25810607 PMC4366910

[7] Nagarajan M, Babu V. 2014. Study on the shifting patterns of non *Candida albicans Candida* in lower respiratory tract infections and evaluation of the CHROM agar in identification of the *Candida species*. J Microbiol Biotechnol Res 1:113–119.

[8] Sobel JD. 2006. The emergence of non-*albicans Candida* species as causes of invasive candidiasis and candidemia. Curr Infect Dis Rep 8:427–433. doi:10.1007/s11908-006-0016-6. 17064635

[9] Clancy CJ, Nguyen MH. 2017. Emergence of *Candida auris*: an international call to arms. Clin Infect Dis 64:141–143. doi:10.1093/cid/ciw696. 27989986

[10] CDC. Global emergence of invasive infections caused by the multidrug-resistant yeast *Candida auris*. CDC, Atlanta, GA.

[11] Drgona L, Khachatryan A, Stephens J, Charbonneau C, Kantecki M, Haider S, Barnes R. 2014. Clinical and economic burden of invasive fungal diseases in Europe: focus on pre-emptive and empirical treatment of *Aspergillus* and *Candida* species. Eur J Clin Microbiol Infect Dis 33:7–21. doi:10.1007/s10096-013-1944-3. 24026863 PMC3892112

[12] Rauseo AM, Coler-Reilly A, Larson L, Spec A. 2020. Hope on the horizon: novel fungal treatments in development. Open Forum Infect Dis 7:ofaa016. doi:10.1093/ofid/ofaa016. 32099843 PMC7031074

[13] Ramachandran R, Shrivastava M, Narayanan NN, Thakur RL, Chakrabarti A, Roy U. 2018. Evaluation of antifungal efficacy of three new cyclic lipopeptides of the class bacillomycin from *Bacillus subtilis* RLID 12.1. Antimicrob Agents Chemother 62:e01457-17. doi:10.1128/AAC.01457-17. 29038271 PMC5740331

[14] Ramchandran R, Ramesh S, A A, Thakur R, Chakrabarti A, Roy U. 2020. Improved production of two anti-*Candida* lipopeptide homologues co-produced by the wild-type *Bacillus subtilis* RLID 12.1 under optimized conditions. Curr Pharm Biotechnol 21:438–450. doi:10.2174/1389201020666191205115008. 31804165

[15] Stein T. 2005. *Bacillus subtilis* antibiotics: structures, syntheses and specific functions. Mol Microbiol 56:845–857. doi:10.1111/j.1365-2958.2005.04587.x. 15853875

[16] Abriouel H, Franz CMAP, Ben Omar N, Gálvez A. 2011. Diversity and applications of *Bacillus* bacteriocins. FEMS Microbiol Rev 35:201–232. doi:10.1111/j.1574-6976.2010.00244.x. 20695901

[17] Mannanov RN, Sattarova RK. 2001. Antibiotics produced by *Bacillus* bacteria. Chem Nat Compd 37:117–123. doi:10.1023/A:1012314516354.

[18] Kwolek-Mirek M, Zadrag-Tecza R. 2014. Comparison of methods used for assessing the viability and vitality of yeast cells. FEMS Yeast Res 14:1068–1079. doi:10.1111/1567-1364.12202. 25154541

[19] Mani-López E, Cortés-Zavaleta O, López-Malo A. 2021. A review of the methods used to determine the target site or the mechanism of action of essential oils and their components against fungi. SN Appl Sci 3:44. doi:10.1007/s42452-020-04102-1.

[20] Ramani R, Ramani A, Wong SJ. 1997. Rapid flow cytometric susceptibility testing of *Candida albicans*. J Clin Microbiol 35:2320–2324. doi:10.1128/jcm.35.9.2320-2324.1997. 9276410 PMC229962

[21] Hao B, Cheng S, Clancy CJ, Nguyen MH. 2013. Caspofungin kills *Candida albicans* by causing both cellular apoptosis and necrosis. Antimicrob Agents Chemother 57:326–332. doi:10.1128/AAC.01366-12. 23114781 PMC3535936

[22] Ludovico P, Sansonetty F, Côrte-Real M. 2001. Assessment of mitochondrial membrane potential in yeast cell populations by flow cytometry. Microbiology (Reading) 147:3335–3343. doi:10.1099/00221287-147-12-3335. 11739765

[23] Jia C, Zhang J, Yu L, Wang C, Yang Y, Rong X, Xu K, Chu M. 2019. Antifungal activity of coumarin against *Candida albicans* is related to apoptosis. Front Cell Infect Microbiol 8:445. doi:10.3389/fcimb.2018.00445. 30662877 PMC6328497

[24] Kourmentza K, Gromada X, Michael N, Degraeve C, Vanier G, Ravallec R, Coutte F, Karatzas KA, Jauregi P. 2021. Antimicrobial activity of lipopeptide biosurfactants against foodborne pathogen and food spoilage microorganisms and their cytotoxicity. Front Microbiol 11:561060. doi:10.3389/fmicb.2020.561060. 33505362 PMC7829355

[25] Mihajlovic M, Lazaridis T. 2010. Antimicrobial peptides in toroidal and cylindrical pores. Biochim Biophys Acta 1798:1485–1493. doi:10.1016/j.bbamem.2010.04.004. 20403332 PMC2885466

[26] Chan LL, Lyettefi EJ, Pirani A, Smith T, Qiu J, Lin B. 2011. Direct concentration and viability measurement of yeast in corn mash using a novel imaging cytometry method. J Ind Microbiol Biotechnol 38:1109–1115. doi:10.1007/s10295-010-0890-7. 20960026

[27] Zhang N, Fan Y, Li C, Wang Q, Leksawasdi N, Li F, Wang S. 2018. Cell permeability and nuclear DNA staining by propidium iodide in basidiomycetous yeasts. Appl Microbiol Biotechnol 102:4183–4191. doi:10.1007/s00253-018-8906-8. 29572560

[28] Yan Y, Tan F, Miao H, Wang H, Cao YY. 2019. Effect of Shikonin against *Candida albicans* biofilms. Front Microbiol 10:1085. doi:10.3389/fmicb.2019.01085. 31156594 PMC6527961

[29] Kuhn DM, George T, Chandra J, Mukherjee PK, Ghannoum MA. 2002. Antifungal susceptibility of *Candida* biofilms: unique efficacy of amphotericin B lipid formulations and echinocandins. Antimicrob Agents Chemother 46:1773–1780. doi:10.1128/AAC.46.6.1773-1780.2002. 12019089 PMC127206

[30] Rodrigues ML. 2018. The multifunctional fungal ergosterol. mBio 9:e01755-18. doi:10.1128/mBio.01755-18. PMC6143734 30228244

[31] Mesa-Arango AC, Scorzoni L, Zaragoza O. 2012. It only takes one to do many jobs: amphotericin B as antifungal and immunomodulatory drug. Front Microbiol 3:286. doi:10.3389/fmicb.2012.00286. 23024638 PMC3441194

[32] Escalante A, Gattuso M, Pérez P, Zacchino S. 2008. Evidence for the mechanism of action of the antifungal phytolaccoside B isolated from *Phytolacca tetramera* Hauman. J Nat Prod 71:1720–1725. doi:10.1021/np070660i. 18816139

[33] Leite MCA, de Brito Bezerra AP, de Sousa JP, Guerra FQS, de Oliveira Lima E. 2014. Evaluation of antifungal activity and mechanism of action of citral against *Candida albicans*. Evid Based Complement Alternat Med 2014:378280. doi:10.1155/2014/378280. 25250053 PMC4163309

[34] Choi H, Cho J, Jin Q, Woo E-R, Lee DG. 2012. Antifungal property of dihydrodehydrodiconiferyl alcohol 9′-*O*-β-d-glucoside and its pore-forming action in plasma membrane of *Candida albicans*. Biochim Biophys Acta 1818:1648–1655. doi:10.1016/j.bbamem.2012.02.026. 22406553

[35] Lee W, Lee DG. 2014. An antifungal mechanism of curcumin lies in membrane-targeted action within *Candida albicans*. IUBMB Life 66:780–785. doi:10.1002/iub.1326. 25380239

[36] Lee H, Woo ER, Lee DG. 2018. Apigenin induces cell shrinkage in *Candida albicans* by membrane perturbation. FEMS Yeast Res 18. doi:10.1093/femsyr/foy003. 29346565

[37] Hollmann A, Matos PM, Augusto MT, Castanho MARB, Santos NC. 2013. Conjugation of cholesterol to HIV-1 fusion inhibitor C34 increases peptide-membrane interactions potentiating its action. PLoS One 8:0060302. doi:10.1371/journal.pone.0060302. PMC3614957 23565220

[38] Matos PM, Gonçalves S, Santos NC. 2008. Interaction of peptides with biomembranes assessed by potential-sensitive fluorescent probes. J Peptide Science 14:407–415. doi:10.1002/psc.1005. 18189333

[39] Cladera J, O’Shea P. 1998. Intramembrane molecular dipoles affect the membrane insertion and folding of a model amphiphilic peptide. Biophys J 74:2434–2442. doi:10.1016/S0006-3495(98)77951-2. 9591669 PMC1299585

[40] Nasir MN, Besson F. 2012. Interactions of the antifungal mycosubtilin with ergosterol-containing interfacial monolayers. Biochim Biophys Acta 1818:1302–1308. doi:10.1016/j.bbamem.2012.01.020. 22306791

[41] Zhang B, Dong C, Shang Q, Han Y, Li P. 2013. New insights into membrane-active action in plasma membrane of fungal hyphae by the lipopeptide antibiotic bacillomycin L. Biochim Biophys Acta 1828:2230–2237. doi:10.1016/j.bbamem.2013.05.033. 23756779

[42] Pina-Vaz C, Sansonetty F, Rodrigues AG, Costa-Oliveira S, Tavares C, Martinez-De-Oliveira J. 2001. Cytometric approach for a rapid evaluation of susceptibility of *Candida* strains to antifungals. Clin Microbiol Infect 7:609–618. doi:10.1046/j.1198-743x.2001.00307.x. 11737085

[43] Green L, Petersen B, Steimel L, Haeber P, Current W. 1994. Rapid determination of antifungal activity by flow cytometry. J Clin Microbiol 32:1088–1091. doi:10.1128/jcm.32.4.1088-1091.1994. 8027319 PMC267192

[44] Chaturvedi V, Ramani R, Pfaller MA. 2004. Collaborative Study of the NCCLS and flow cytometry methods for antifungal susceptibility testing of *Candida albicans*. J Clin Microbiol 42:2249–2251. doi:10.1128/JCM.42.5.2249-2251.2004. 15131203 PMC404628

[45] Ramani R, Chaturvedi V. 2000. Flow cytometry antifungal susceptibility testing of pathogenic yeasts other than *Candida albicans* and comparison with the NCCLS broth microdilution test. Antimicrob Agents Chemother 44:2752–2758. doi:10.1128/AAC.44.10.2752-2758.2000. 10991856 PMC90147

[46] Tabbene O, Kalai L, Ben Slimene I, Karkouch I, Elkahoui S, Gharbi A, Cosette P, Mangoni ML, Jouenne T, Limam F. 2011. Anti-*Candida* effect of bacillomycin D-like lipopeptides from *Bacillus subtilis* B38. FEMS Microbiol Lett 316:108–114. doi:10.1111/j.1574-6968.2010.02199.x. 21204933

[47] Belenky P, Camacho D, Collins JJ. 2013. Fungicidal drugs induce a common oxidative-damage cellular death pathway. Cell Rep 3:350–358. doi:10.1016/j.celrep.2012.12.021. 23416050 PMC3656588

[48] González-Párraga P, Sánchez-Fresneda R, Zaragoza Ó, Argüelles JC. 2011. Amphotericin B induces trehalose synthesis and simultaneously activates an antioxidant enzymatic response in *Candida albicans*. Biochim Biophys Acta 1810:777–783. doi:10.1016/j.bbagen.2011.04.012. 21570449

[49] Mesa-Arango AC, Trevijano-Contador N, Román E, Sánchez-Fresneda R, Casas C, Herrero E, Argüelles JC, Pla J, Cuenca-Estrella M, Zaragoza O. 2014. The production of reactive oxygen species is a universal action mechanism of amphotericin B against pathogenic yeasts and contributes to the fungicidal effect of this drug. Antimicrob Agents Chemother 58:6627–6638. doi:10.1128/AAC.03570-14. 25155595 PMC4249417

[50] Čáp M, Váchová L, Palková Z. 2012. Reactive oxygen species in the signaling and adaptation of multicellular microbial communities. Oxid Med Cell Longev 2012:976753. doi:10.1155/2012/976753. 22829965 PMC3395218

[51] Delattin N, de Brucker K, Vandamme K, Meert E, Marchand A, Chaltin P, Cammue BPA, Thevissen K. 2014. Repurposing as a means to increase the activity of amphotericin B and caspofungin against *Candida albicans* biofilms. J Antimicrob Chemother 69:1035–1044. doi:10.1093/jac/dkt449. 24284780

[52] de Brucker K, Cammue BPA, Thevissen K. 2011. Apoptosis-inducing antifungal peptides and proteins. Biochem Soc Trans 39:1527–1532. doi:10.1042/BST0391527. 21936846

[53] Hwang I, Lee J, Lee DG. 2011. Indole-3-carbinol generates reactive oxygen species and induces apoptosis. Biol Pharm Bull 34:1602–1608. doi:10.1248/bpb.34.1602. 21963502

[54] Yu Q, Zhang B, Li J, Zhang B, Wang H, Li M. 2016. Endoplasmic reticulum-derived reactive oxygen species (ROS) is involved in toxicity of cell wall stress to *Candida albicans*. Free Radic Biol Med 99:572–583. doi:10.1016/j.freeradbiomed.2016.09.014. 27650297

[55] Wang Q, Pan L, Han Y, Zhou Z. 2022. Antimicrobial mechanisms of enterocin CHQS against *Candida albicans*. Curr Microbiol 79:191. doi:10.1007/s00284-022-02878-6. 35552837

[56] Neto JBA, da Silva CR, Neta MAS, Campos RS, Siebra JT, Silva RAC, Gaspar DM, Magalhães HIF, de Moraes MO, Lobo MDP, Grangeiro TB, Carvalho TSC, Diogo EBT, da Silva Júnior EN, Rodrigues FAR, Cavalcanti BC, Júnior HVN. 2014. Antifungal activity of naphthoquinoidal compounds *in vitro* against fluconazole-resistant strains of different *Candida* species: a special emphasis on mechanisms of action on *Candida tropicalis*. PLoS One 9:e93698. doi:10.1371/journal.pone.0093698. 24817320 PMC4015898

[57] Ramachandran R, Ramesh S, Ramkumar S, Chakrabarti A, Roy U. 2018. Calcium alginate bead-mediated enhancement of the selective recovery of a lead novel antifungal bacillomycin variant. Appl Biochem Biotechnol 186:917–936. doi:10.1007/s12010-018-2778-3. 29797296

[58] CLSI. 2008. Reference method for broth dilution antifungal susceptibility testing of yeasts, approved standard, 3rd ed. M27-A3. CLSI, Wayne, PA.

[59] Clancy CJ, Huang H, Cheng S, Derendorf H, Nguyen MH. 2006. Characterizing the effects of caspofungin on *Candida albicans*, *Candida parapsilosis*, and *Candida glabrata* isolates by simultaneous time-kill and postantifungal-effect experiments. Antimicrob Agents Chemother 50:2569–2572. doi:10.1128/AAC.00291-06. 16801448 PMC1489803

[60] Mosmann T. 1983. Rapid colorimetric assay for cellular growth and survival: application to proliferation and cytotoxicity assays. J Immunol Methods 65:55–63. doi:10.1016/0022-1759(83)90303-4. 6606682

[61] Fischer ER, Hansen BT, Nair V, Hoyt FH, Dorward DW. 2012. Scanning electron microscopy. Curr Protoc Microbiol Chapter 2:Unit 2B.2. doi:10.1002/9780471729259.mc02b02s25. PMC3352184 22549162

[62] Semis R, Kagan S, Berdicevsky I, Polacheck I, Segal E. 2013. Mechanism of activity and toxicity of Nystatin-Intralipid. Med Mycol 51:422–431. doi:10.3109/13693786.2012.731712. 23088298

[63] Pina-Vaz C, Sansonetty F, Rodrigues AG, Costa-De-Oliveira S, Martinez-De-Oliveira J, Fonseca AF. 2001. Susceptibility to fluconazole of *Candida* clinical isolates determined by FUN-1 staining with flow cytometry and epifluorescence microscopy. J Med Microbiol 50:375–382. doi:10.1099/0022-1317-50-4-375. 11289523

[64] Aguiar FLL, Santos NC, de Paula Cavalcante CS, Andreu D, Baptista GR, Gonçalves S. 2020. Antibiofilm activity on *Candida albicans* and mechanism of action on biomembrane models of the antimicrobial peptide Ctn[15–34]. Int J Mol Sci 21:8339. doi:10.3390/ijms21218339. 33172206 PMC7664368

[65] Chung KT, Kwang WB, Hyung IS, Jae KK, Yong JJ. 1989. Conditions for protoplast formation and fusion of the killer yeast. Korean J Microbiol 27:422–429.

[66] Seyedjavadi SS, Khani S, Eslamifar A, Ajdary S, Goudarzi M, Halabian R, Akbari R, Zare-Zardini H, Imani FA, Amani J, Razzaghi-Abyaneh M. 2020. The antifungal peptide MCh-AMP1 derived from *Matricaria chamomilla* inhibits *Candida albicans* growth via inducing ROS generation and altering fungal cell membrane permeability. Front Microbiol 10:3150. doi:10.3389/fmicb.2019.03150. 32038583 PMC6985553

[67] da Silva CR, de Andrade Neto JB, de Sousa Campos R, Figueiredo NS, Sampaio LS, Magalhães HIF, Cavalcanti BC, Gaspar DM, de Andrade GM, Lima ISP, de Barros Viana GS, de Moraes MO, Lobo MDP, Grangeiro TB, Nobre HV. 2014. Synergistic effect of the flavonoid catechin, quercetin, or epigallocatechin gallate with fluconazole induces apoptosis in *Candida tropicalis* resistant to fluconazole. Antimicrob Agents Chemother 58:1468–1478. doi:10.1128/AAC.00651-13. 24366745 PMC3957875

