# Supplementary material for: Functional Characterization of a Bacillus-Derived Novel Broad-Spectrum Antifungal Lipopeptide Variant against Candida tropicalis and Candida auris and Unravelling Its Mode of Action
Source: Microbiol Spectr. 2023 Feb 6;11(2):e01583-22. doi: 10.1128/spectrum.01583-22 (PMC10100908; doi:10.1128/spectrum.01583-22)
Supplement: Supplemental file 1 — Supplemental material. Download spectrum.01583-22-s0001.pdf, PDF file, 2.2 MB [file spectrum.01583-22-s0001.pdf]

Supplementary Materials

1. Antifungal susceptibility testing with AF_4 and AMB against *Candida* non-*albicans* strains:

Table S1: MICs and MFCs of AF_4 against *Candida tropicalis* and *Candida auris* strains, *C. albicans* SC5314 and *C. glabrata* as reference strains.

**Organism**	**AF_4**		**AMB**	
	**MIC (mg/L)**	**MFC (mg/L)**	**MIC (mg/L)**	**MFC (mg/L)**
***C. albicans* SC5314**	**4-8**	**8.0**	**0.5**	**0.5**
***C. glabrata* ATCC 2001**	4.0	4.0	1.0	n.d.
***C. tropicalis* ATCC 750**	4.0	4.0	1.0	1.0
***C. tropicalis* P-2908**	4.0	4.0	n.d.	n.d.
***C. tropicalis* P-36429**	4.0	4.0	1.0	1.0
***C. tropicalis* P-36020**	4.0	4.0	2.0	2.0
***C. tropicalis* IL- 4026**	8.0	8.0	2.0	4.0
***C. tropicalis* IL-4028**	8.0	8.0	2.0	4.0
***C. auris* IL-3331**	4.0	4.0	2.0	2.0
***C. auris* IL-3301**	8.0	8.0	4.0	4.0
***C. auris* IL-3297**	2.0	4.0	1.0	1.0
***C. auris* IL-3298**	2.0	4.0	1.0	1.0

n.d. – not determined

2. Optimised protocol for the production of Antifungal lipopeptides

B. subtilis is grown for 60h at 110rpm and 30°C. Crude peptide mixture has been obtained by acid precipitation from the cell-free supernatant which is followed by fractionation by silica gel-based adsorption chromatography using a chloroform-methanol gradient. Fractions are tested for activity against *C. albicans* SC5314 by spot-on-lawn assay. The fractions that show inhibition zones are separated and purified into five variants/homologues by reverse phase semi-preparative scale HPLC.

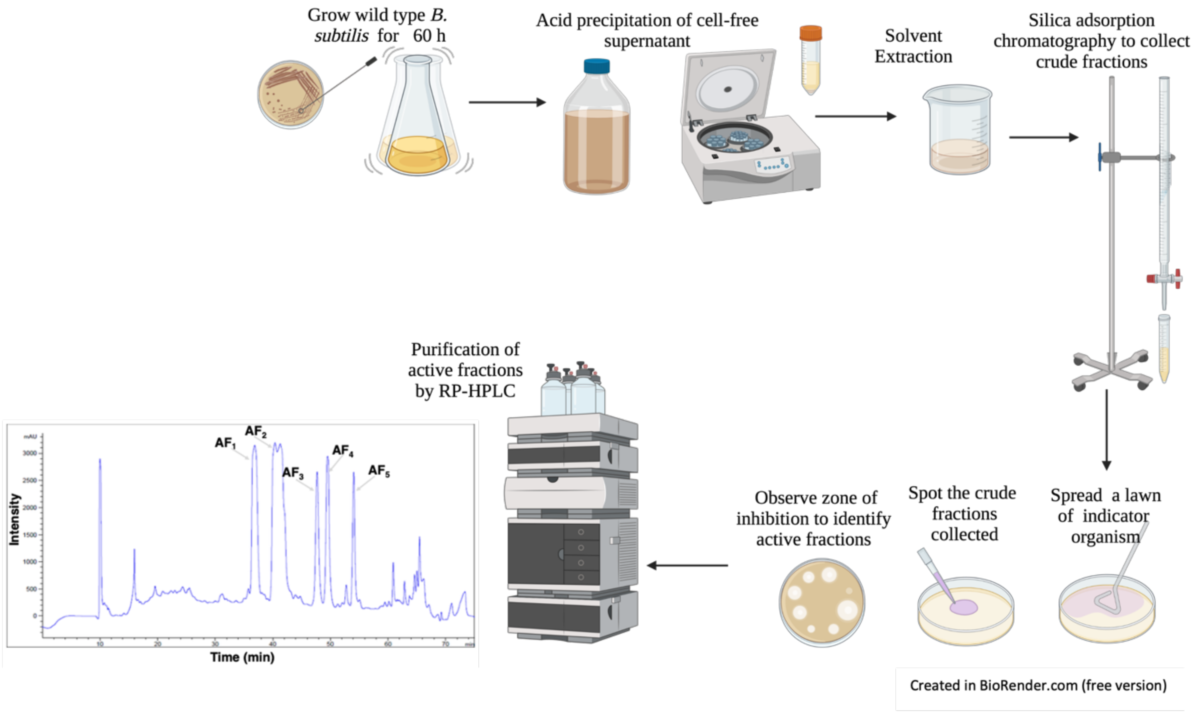

Fig. S1 Graphical representation of the optimised protocol used for antifungal lipopeptide extraction and purification.

3. Mass spectrometry analysis by Electrospray Ionization Fourier Transform Ion Cyclotron Resonance Mass Spectrometry (ESI-FT-ICR-MS):

Analysis was performed on an ESI-FT-ICR mass spectrometer (SolariX, Bruker) in a positive ion mode at a concentration of 9.3 µM in acetonitrile-water-formic acid [50:50:0.1 (v/v/v)]. Analysis of spectra and elemental compositions were generated using the data analysis (X-mass acquisition) software vs 4.4 (Bruker).

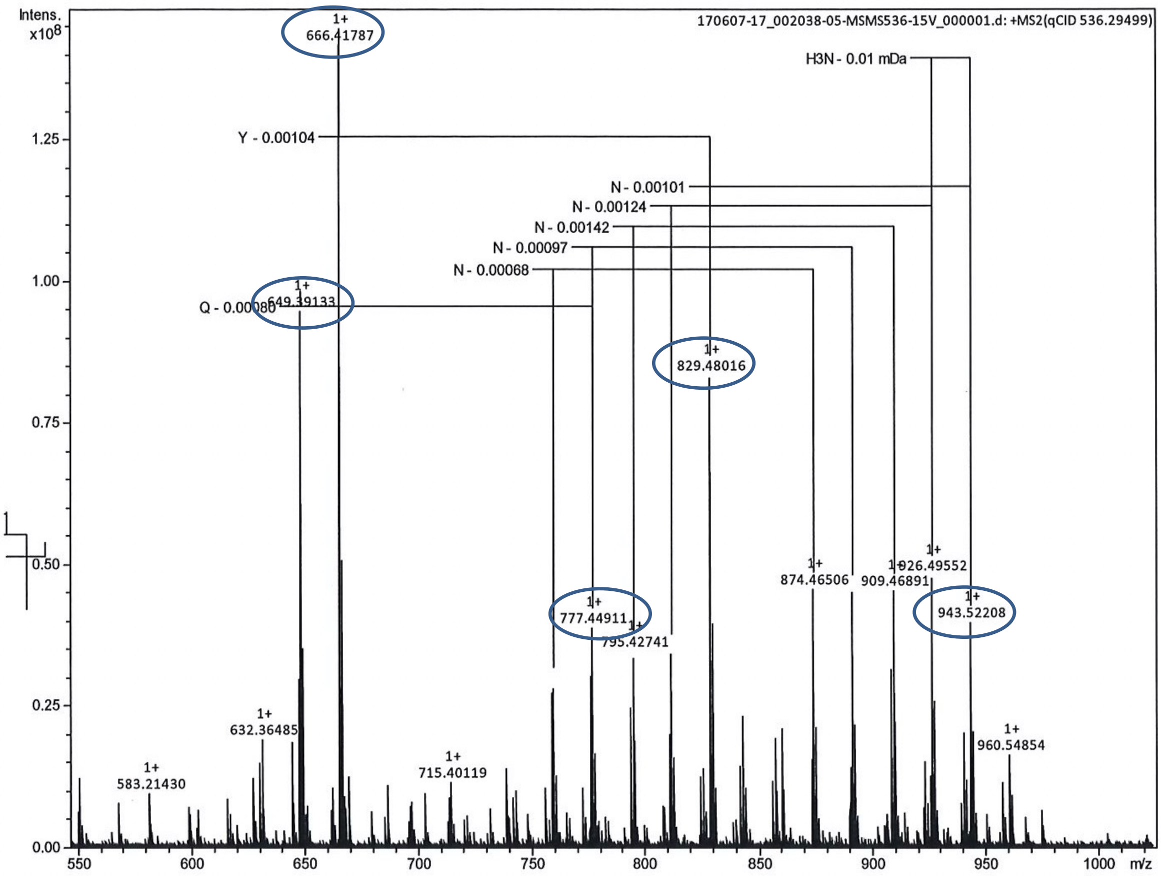

Fig. S2 CID profile of ions after ESI-FT-ICR MS/MS of AF_4

The ESI-Fourier Transform ion Cyclotron Resonance (ESI-FT-ICR MS/MS) was performed for the purified AF4 to determine the entire sequence. In the collision-induced dissociation (CID) profile of the ion at m/z 1071.58 (Fig. S5), connected losses of ions were spotted by the fragment's m/z 1054.5 (loss of H_2O- not seen in this graph), 943.5 (1071.58-943.5= 128 (Q)), 829.5 (Q-N), 666.4 (Q-N-Y), 569.5 (Q-N-Y-P) and 455.3 (Q-N-Y-P-N). The MS/MS spectrum revealed the m/z 128.06 (777.449-649.391) corresponding to Gln (Q), m/z 114.04 (943.52-829.48) corresponding to Asn (N), and the m/z 163.07 (829.48-666.41) to Tyr (Y).

4. Scanning electron microscopy:

C. tropicalis and *C. auris* cells treated with AF_4 at 8 mg/L to observe the effect of lipopetide on cell surface.

A

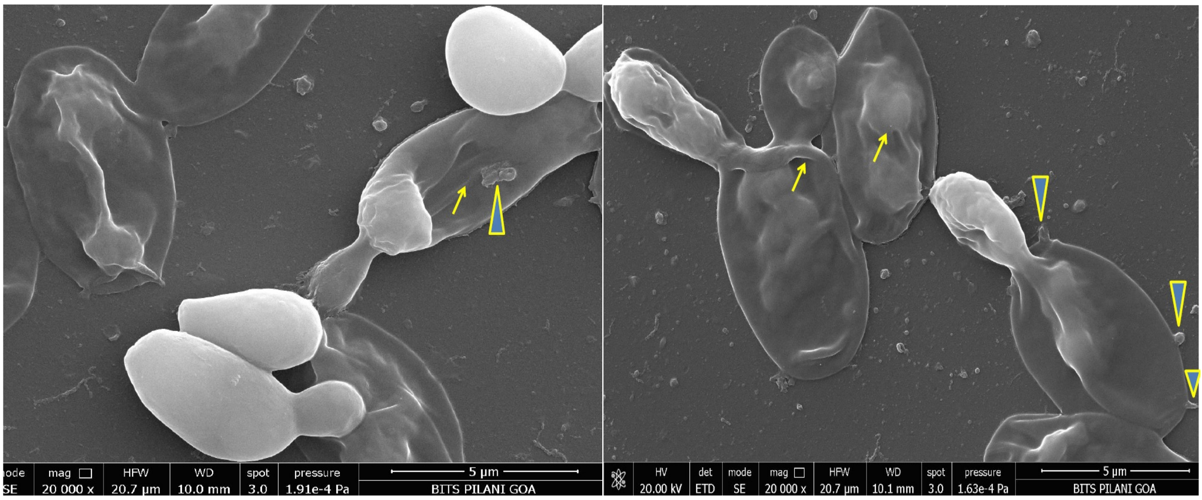

B

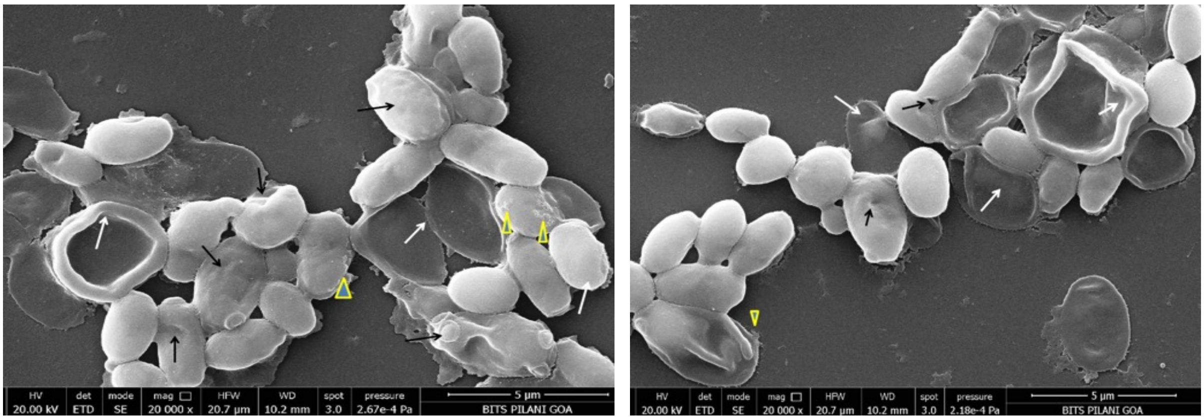

Fig. S3 (A) Left panel. AF_4 (8 mg/L)-treated *C. tropicalis* cells exhibit highly deformed surfaces (yellow arrows) with occasional distinct membrane blebs or protrusions indicated by yellow arrowheads. Right panel. Yellow arrows indicate the completion of cell collapses with indentations and loss of shapes. **(B)** Left panel. AF_4 (8 mg/L)-treated *C. auris* cells exhibit highly dimpled and rugged surfaces (black arrows) with occasional membrane blebs indicated by yellow-bordered arrowheads. White arrows indicate the presence of completely collapsed cells. Aggregated damaged cells with bud-scars are indicated by black arrows. Right panel. Aggregated damaged cells with bud-scars are strewn across the field. Membrane protrusions are shown by yellow-bordered arrowheads. The scale bar shown is 5 µm.

5. Extraction and quantification of ergosterol

Ergosterol from cells of *C. tropicalis* and *C. auris* were extracted using alcoholic KOH and *n*-hexane(de Aguiar Cordeiro et al., 2014) after treating the cells with AF_4(8 mg/L) and AMB(MIC values) overnight. The amount of ergosterol extracted across treatments was determined from absorbance readings taken at 295 nm and compared with the Ergosterol standard (Sigma-Aldrich, USA). Figure S4 represents the percentage of ergosterol present in the samples after extraction into *n*-hexane across treatments. The reduced recovery of ergosterol suggests the absence of growth in treated cells and presents the extent of antifungal activity of AF_4.

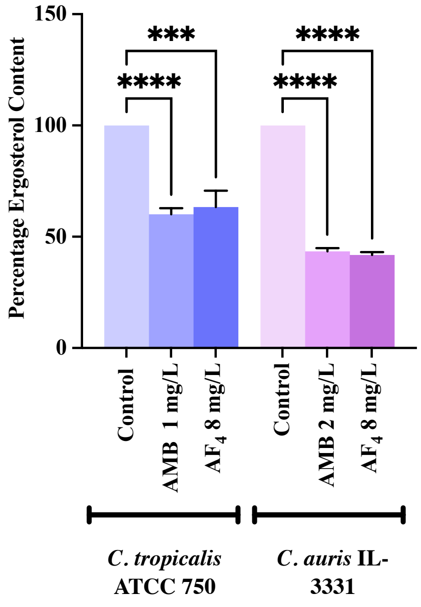

Figure S4. Effect of AF_4 on ergosterol content of fungal cell membranes of *C. tropicalis* and *C. auris*. Percentage ergosterol in cells treated with AMB at respective MIC values and AF_4 at 8 mg/L.

6. PI uptake analysis by flow cytometry

All events graphs showing the population of cells which were used for analysis.

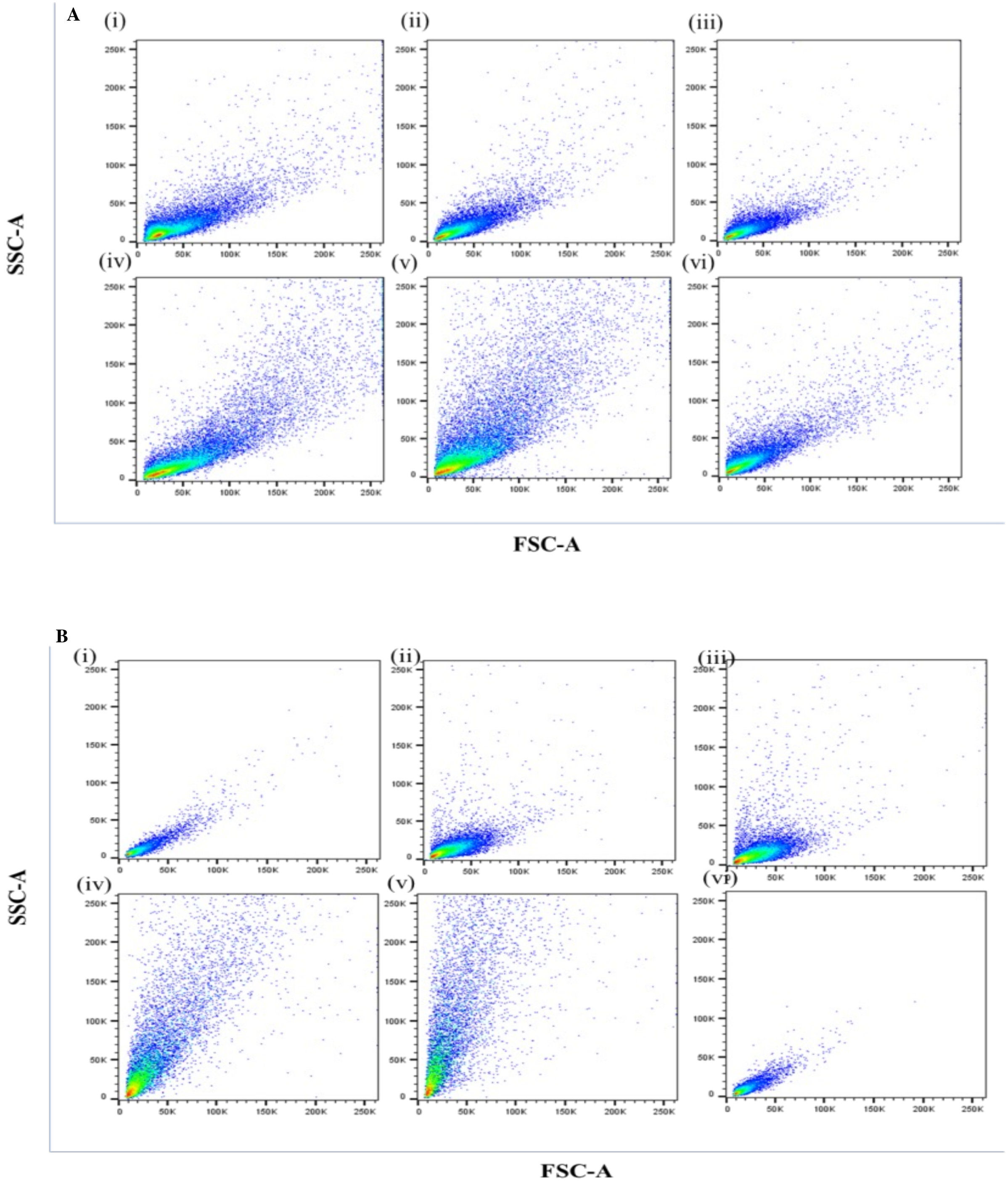

Fig. S5 Pseudocolour plots of FCM analysis. (**A**) FC pseudocolor plots showing all events graphs of *C. tropicalis* ATCC 750 cells. Panels are (i) Untreated cells, (ii) AMB (2 mg/L), (iii) AMB (4 mg/L) and (iv) AF_4 (8 mg/L), (v) AF_4 (16 mg/L) and (vi) 70% ethanol-treated. (**B)** FC pseudocolor plots showing all events graphs of *C. auris* IL-3331 cells. Panels are (i) Untreated

cells, (ii) AMB (4 mg/L), (iii) AMB (8 mg/L) and (iv) AF_4 (8 mg/L), (v) AF_4 (16 mg/L) and (vi) 70% ethanol-treated.

7. Flow cytometric analysis of cell size

The cell size and granularity/complexity of untreated cells and cells treated with AF_4 is inferred from the distribution of the contour plot of FSC; forward scatter and granularity SSC; side scatter. The cell size and granularity/complexity as inferred from the top right quadrant of the panel (iii).

A

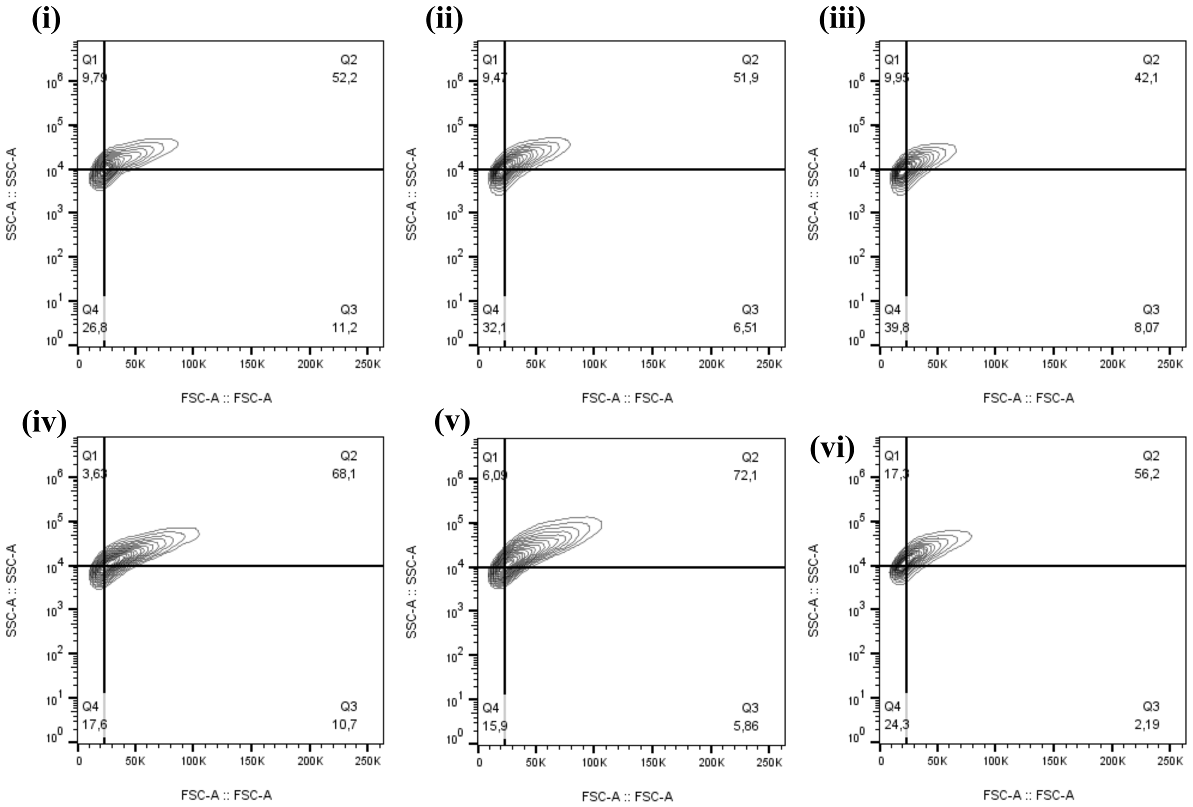
(i)
Q1 9,79
Q2 52,2
Q4 26,8
Q3 11,2
(ii)
Q1 9,47
Q2 51,9
Q4 32,1
Q3 6,51
(iii)
Q1 9,95
Q2 42,1
Q4 39,8
Q3 8,07
(iv)
Q1 3,63
Q2 68,1
Q4 17,6
Q3 10,7
(v)
Q1 6,09
Q2 72,1
Q4 15,9
Q3 5,86
(vi)
Q1 17,3
Q2 56,2
Q4 24,3
Q3 2,19
SSC-A :: SSC-A
FSC-A :: FSC-A
0 50K 100K 150K 200K 250K

B

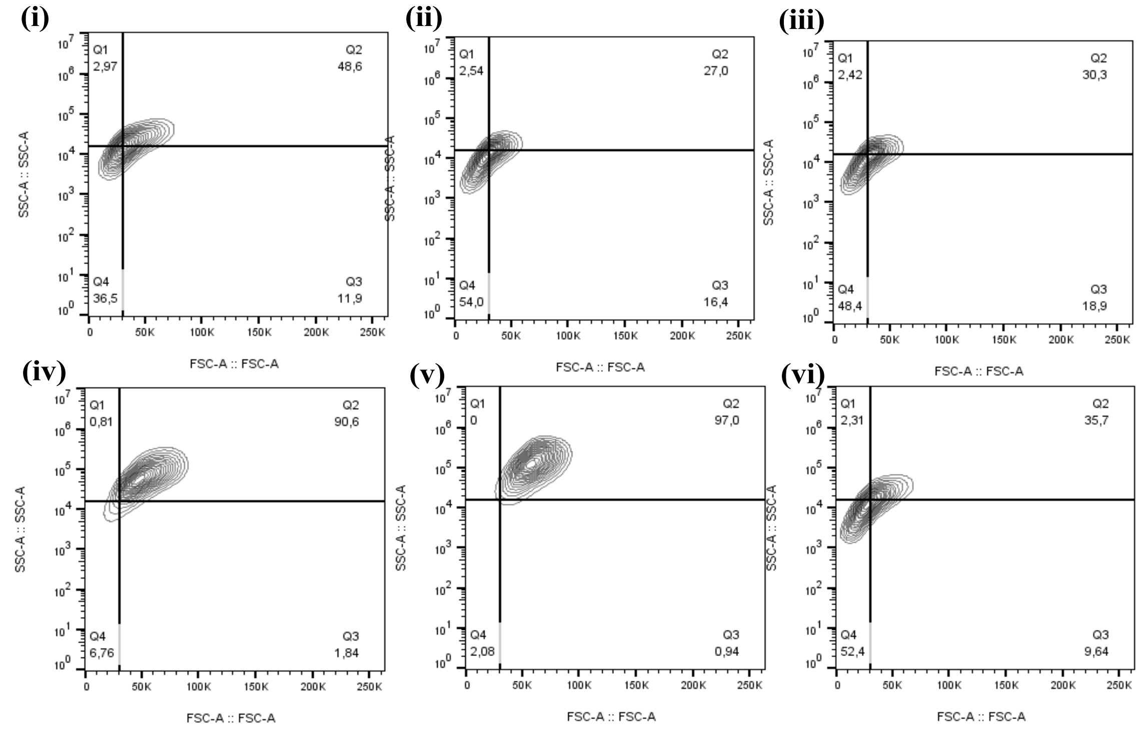
(i)
Q1 2,97
Q2 48,6
Q4 36,5
Q3 11,9
(ii)
Q1 2,54
Q2 27,0
Q4 54,0
Q3 16,4
(iii)
Q1 2,42
Q2 30,3
Q4 48,4
Q3 18,9
(iv)
Q1 0,81
Q2 90,6
Q4 6,76
Q3 1,84
(v)
Q1 0
Q2 97,0
Q4 2,08
Q3 0,94
(vi)
Q1 2,31
Q2 35,7
Q4 52,4
Q3 9,64
SSC-A :: SSC-A
FSC-A :: FSC-A
0 50K 100K 150K 200K 250K

Fig. S6 Flow cytometric analysis of cell size: (A) ***C. tropicalis* ATCC 750** FSC; forward scatter and granularity SSC; side scatter after treatment. The cell size and granularity/complexity as inferred from the top right quadrant of panel (iii). The percentage depicted is indicative of cells falling in each quadrant Q1: FSC-A−, SSC-A+ Q2: FSC-A+, SSC-A+ Q3: FSC-A+ SSC-A− Q4: FSC-A- SSC-A−. Panels are (i) Untreated cells, (ii) AMB (2 mg/L), (iii) AMB (4 mg/L) and (iv)AF_4 (8 mg/L), (v) AF_4 (16 mg/L) and (vi) 70% ethanol-treated. **(B)** ***C. auris* IL-3331** cell size FSC; forward scatter and granularity SSC; side scatter after treatment. The cell size and granularity/complexity as inferred from the top right quadrant of panel (iii). The percentage depicted is indicative of cells falling in each quadrant Q1: FSC-A−, SSC-A+ Q2: FSC-A+, SSC-A+ Q3: FSC-A+ SSC-A− Q4: FSC-A- SSC-A−. Panels are (i) Untreated cells, (ii) AMB (2 mg/L), (iii) AMB (4 mg/L) and (iv) AF_4 (8 mg/L), (v) AF_4 (16 mg/L) and (vi) 70% ethanol-treated.

8. **Rh123 as a live/dead probe**

Cells stained with Rh123 can be differentiated into live and dead cells based on their distribution along the x-axis. Cells present in gate P7 are considered live cells, whereas those in gate P8 are considered to have undergone cell death resulting in excessive and diffuse fluorescence of Rh123 as it is unable to localise to the mitochondrial membrane.

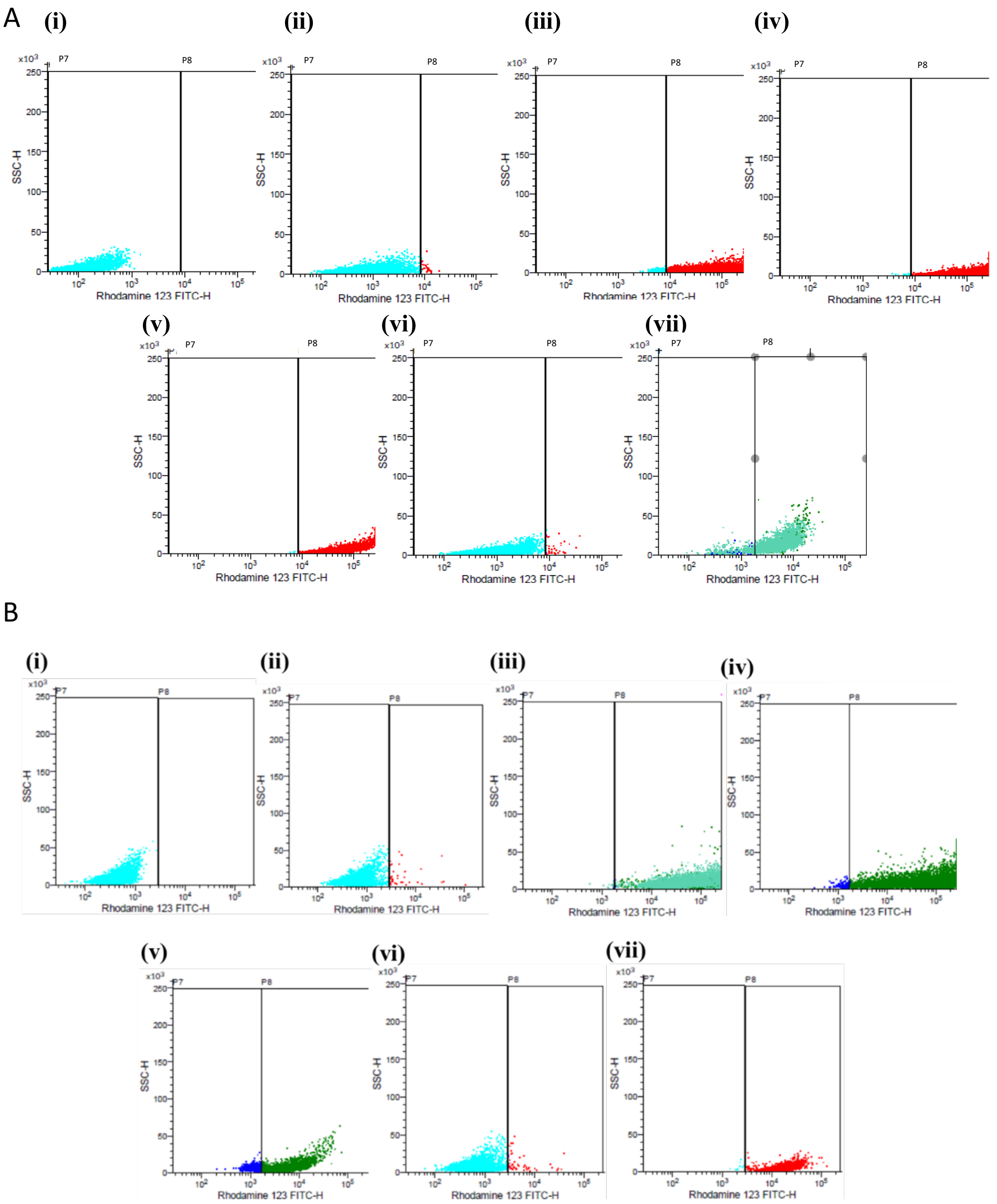

Fig. S7 Rh123 staining to differentiate between live and dead cell. (A) FC histograms of *C. tropicalis* ATCC 750 cells stained with Rh123 analysed using FITC filter. Panels are (i) Unstained cells, (ii) Untreated cells, (iii) AF_4 (8 mg/L), (iv) AF_4 (16 mg/L) and (v) 70% ethanol-treated, (vi) Sodium azide (40 mM) treatment (vii) 121°C treatment. 10000 events analysed. **(B)** FC histograms of *C. auris* IL-3331 cells stained with Rh123 analysed using FITC filter. Panels are of SSC-H vs. Rh123-H (i) Unstained cells, (ii) Untreated Cells, (iii) AF_4 (8 mg/L),

(iv) AF_4 (16 mg/L) and (v) 70% ethanol-treated, (vi) Sodium azide (40 mM) treatment (vii) 121°C treatment. 10000 events analysed.

9. Schematic representation of the mode of action

The probable mode of action of AF_4 against *Candida* cells. AF_4 exerts antifungal activity primarily by binding to outer leaflet of plasma membrane, ergosterol binding, damaging cell membrane structure, enhancing cell membrane permeability, intracellular ROS generation, mitochondrial membrane dysfunction and nuclear damage.

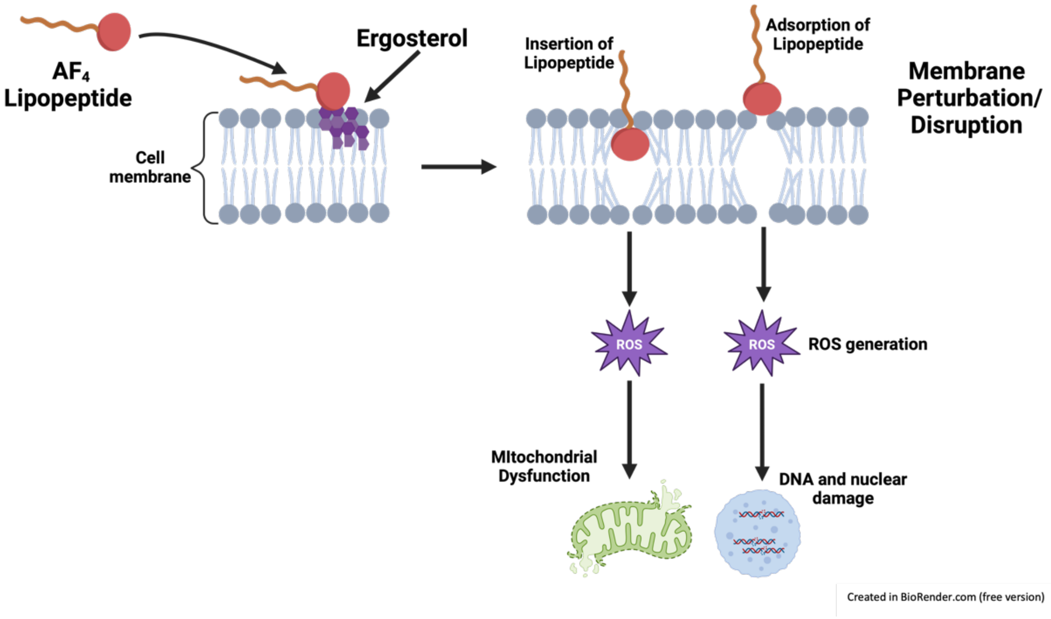

Fig. S8 Schematic representation of the proposed mode of action of AF_4 lipopeptide on *Candida* cells.
